# The ML1Nx2 Phosphatidylinositol 3,5-Bisphosphate Probe Shows Poor Selectivity in Cells

**DOI:** 10.1371/journal.pone.0139957

**Published:** 2015-10-13

**Authors:** Gerald R. V. Hammond, Shunsuke Takasuga, Takehiko Sasaki, Tamas Balla

**Affiliations:** 1 Department of Cell Biology, University of Pittsburgh School of Medicine, Pittsburgh, PA, United States of America; 2 Program in Developmental Neuroscience, Eunice Kennedy Shriver National Institute of Child Health and Human Development, National Institutes of Health, Bethesda, MD, 20892, United States of America; 3 Department of Medical Biology, Graduate School of Medicine, Akita University, Akita, Japan; 4 Research Center for Biosignal, Akita University, Akita, 010–8543, Japan; Queen Mary University of London, Blizard Institute, UNITED KINGDOM

## Abstract

Phosphatidylinositol (3,5)-bisphosphate (PtdIns(3,5)*P*_2_) is a quantitatively minor phospholipid in eukaryotic cells that plays a fundamental role in regulating endocytic membrane traffic. Despite its clear importance for cellular function and organism physiology, mechanistic details of its biology have so far not been fully elucidated. In part, this is due to a lack of experimental tools that specifically probe for PtdIns(3,5)*P*_2_ in cells to unambiguously identify its dynamics and site(s) of action. In this study, we have evaluated a recently reported PtdIns(3,5)*P*_2_ biosensor, GFP-ML1Nx2, for its veracity as such a probe. We report that, in live cells, the localization of this biosensor to sub-cellular compartments is largely independent of PtdIns(3,5)*P*_2_, as assessed after pharmacological, chemical genetic or genomic interventions that block the lipid’s synthesis. We therefore conclude that it is unwise to interpret the localization of ML1Nx2 as a true and unbiased biosensor for PtdIns(3,5)*P*_2_.

## Introduction

The phospholipid phosphatidylinositol (PtdIns) plays a unique role in membrane function, through reversible phosphorylation at three positions on its inositol head group, creating a family of seven unique bioactive lipids that direct protein activity [1]. The scarcest of these lipids in most cells, and the last to be discovered, is PtdIns(3,5)*P*_2_ [2]. Despite being a quantitatively minor lipid, PtdIns(3,5)*P*_2_ is now appreciated as a functionally crucial molecule in the endocytic pathway [3].

PtdIns(3,5)*P*_2_ is synthesized from the more abundant PtdIns3*P* by the 5-kinase Fab1p in yeast [4,5] and its homologue PIKfyve in animals [6], and the reaction is reversed by the Sac-phosphatase domain containing Fig 4/Sac3 [7]. Interestingly, in yeast and animals these enzymes exist as a single complex with their mutual regulator Vac14/ArPIKfyve [8–12]. Degradation of PtdIns(3,5)*P*_2_ by a 3-phosphatase activity of myotubularin-family phosphatases is also possible [13], although whether the pathway actually operates to accumulate another scarce inositol lipid, PtdIns5*P*, is still hotly debated [14].

Most studies of the function of PtdIns(3,5)*P*_2_ come from either genetic inactivation of the Fab1p/PIKfyve complex [8–12,15] or pharmacological inhibition of the enzyme [16].The overarching conclusion from such studies is that PtdIns(3,5)*P*_2_ controls endocytic trafficking, principally between endosomes and lysosomes (vacuoles in yeast) and the Golgi [3]. Such a fundamental cellular role for the lipid combined with a single enzyme responsible for the terminal step of its synthesis makes for profound impacts on organism function after inactivation of the PIKfyve gene, leading to embryonic lethality in mice [17,18]. Tissue-specific mutations in PIKfyve complex components lead to a number of pathological conditions in mice, including neurodegeneration [19,20] and defective glucose homeostasis [21], with implications for the lipid’s role in human disease.

Despite such clear and crucial roles for cellular and organismal physiology, mechanistic detail as to how PtdIns(3,5)*P*_2_ executes these functions is still emerging. Several effector proteins of the lipid have been identified [22], but unambiguously defining the lipid’s mechanism of action requires tools to probe its cellular localization and function with respect to these effector proteins. This stems from the fact that whereas protein-lipid interactions may be necessary for protein localization and function, they are often not sufficient in themselves, and additional factors may further restrict their distribution [23].

A recently identified PtdIns(3,5)*P*_2_ effector protein is the mucolipin cation channel TRPML1 [24,25]. The apparent specificity of the channel-lipid interaction led to the isolation of the soluble N-terminus (ML1N) as the lipid interacting domain, and its subsequent engineering into a tandem dimer (ML1Nx2) with increased avidity for the lipid for use as a specific probe for PtdIns(3,5)*P*_2_ in living cells [26]. This represented a potential break-through for the field, and unambiguously assigned PtdIns(3,5)*P*_2_ to endosomes and lysosomes [26].

We recently characterized a live-cell biosensor for another inositol lipid, PtdIns4*P*, and localized this lipid to endosomes and lysosomes as well [27]. We were therefore interested to study whether these two inositol lipids might have overlapping localizations and functions. However, in the course of these studies, we discovered strong evidence that the ML1Nx2 probe’s localization to endosomes and lysosomes is largely independent of PtdIns(3,5)*P*_2_. We describe the results of these studies herein, and sound a note of caution when interpreting the distribution of ML1Nx2 as an indicator for the lipid’s localization or abundance. It seems that the ultimate cellular distribution of PtdIns(3,5)*P*_2_ has yet to be directly observed.

## Materials and Methods

### Plasmids and Reagents

GFP-ML1Nx2 was a kind gift from Dr. Haoxing Xu (University of Michigan, Ann Arbor, MI, USA). Fluorescent protein conjugated P4M, PH-PLCδ1-mCherry, mTurquoise-FYVE-EEA1, iRFP-FRB-Rab5 and -Rab7, mCherry-FKBP-MTM1 were as described previously [27]. mTurquoise-Rab7 was modified from an mCherry-Rab7 construct [28] kindly donated by R. Lodge (Institut de Recherches Cliniques de Montreal, Montreal, Quebec, Canada) to replace mCherry with mTurquoise2 [29]. Lamp1-mRFP [30] was a kind gift from Marko Jovic (UDC, Washington D.C., USA).

Rapamycin was from Life Technologies, wortmannin and YM201636 were from Selleckcem. We noted that even after storing fresh single use aliquots of YM201636 dissolved in DMSO at –80°C, the compound lost activity over a period of three months. All data presented are from stocks of the compound dissolved in DMSO within 2 weeks of preparation. All other reagents were obtained from Sigma.

### Cell Culture and Transfection

COS-7 cells (ATCC CRL-1651) were grown in high-glucose Dulbecco’s minimum essential medium supplemented with 10% fetal bovine serum and penicillin/streptomycin (Life technologies). They were passaged twice per week by enzymatic dissociation using TrypLE express (Life Technologies) and dilution. Cells seeded in 29 mm glass bottom dishes (CellVis) were transfected at ~50% confluency in 1 ml Opti-MEM (Life Technologies) pre-complexed for 20 min with 3 μg Lipofectamine2000 from a 1 mg/ml stock solution (Life Technologies) and 1 μg total plasmid DNA. Ratios of different plasmids were empirically determined to reach optimum transfection efficiency of all plasmids.

### Live Cell Imaging

Imaging was performed on a Zeiss 780 confocal laser scanning confocal microscope using a 63x, 1.4 NA plan-apochromatic objective lens mounted on a Zeiss Axio Observer stand. Fluorophores, excitation laser lines and spectral detection windows and detectors were respectively as follows: CFP, 405 nm excitation, 450–490 nm detection, Quasar GaSP PMT array; GFP, 488 nm excitation, 508–535 nm detection, Quasar GaSP PMT array; mCherry/mRFP, 561 nm excitation, 578–649 nm detection, Quasar GaSP PMT array; iRFP, 633 nm excitation, 650–748 nm detection, PMT. CFP was acquired simultaneously with mCherry, as were GFP and iRFP; where all four are detected, sequential imaging between the two pairs was performed to eliminate cross-talk. Images from multiple positions were recorded in series using a motorised stage at the time interval indicated in each Fig.

The cells were bathed in 800 μl of HEPES-containing high-glucose phenol red-free Dulbecco’s modified essential medium (Life Technologies). As indicated in Fig legends, drug additions were made in 200 μl medium at 5x final concentration. An automated focusing system (Definite Focus, Zeiss) was used to maintain the confocal plane during time-lapse imaging.

### Image Analysis

All Image analysis was performed in Fiji [31]. Co-localization analysis used calculation of the normalized mean deviation product (nMDP), a co-localization method that allows quantitative analysis of two channels with an image-based presentation [32], and was calculated using a custom-written Fiji macro as described previously [27]. Briefly, pixel intensity for both channels in a region of interest (ROI) encompassing the whole cell is normalized to the mean intensity and set to a range between 1 and –1 (that is, mean = 0, brightest pixel = 1, dimmest pixel = –1). The two normalized images are then multiplied together; pixels with correlating intensities (both towards larger more positive or negative values) produce high, positive values (yellow in the images), whereas regions that anti-correlate (bright in one channel verses dim in another) tend towards smaller or even negative pixel values (green in images). For clarity in displayed images, pixels that were dim in both channels (i.e. < 0 and therefore a positive product) are set to 0 (black), although this is performed after calculating the image’s nMDP score.

Image intensity analysis was also performed as described in detail before [27]. Briefly, normalized intensity was calculated for each cell by normalizing each pixel to the mean for a region of interest (ROI) encompassing the whole cell. The mean pixel intensity for endosomal or Lamp1-positive compartments was then calculated by generating an automated binary mask of these compartments based on images of the Rab5/7 or lamp-1 markers at each time-point. This mask is generated via an elaborate auto-thresholding technique utilizing a-trous wavelet decomposition of these images [33].

To select example images for Figs, all the data was ranked by the appropriate metric (nMDP score or compartment-specific normalized pixel intensity) and examples showing clear morphology, high signal/noise and a score close to the median were selected. In all cases, image scores fall within the 25–75 percentile range. Graphs show the data from every cell analysed in all experiments; they were constructed in Prism6 (Graphpad) and statistical analysis was performed in the same software as indicated for each Fig.

### Pikfyve-deficient ES Cells

Pikfyve-deficient murine embryonic stem (ES) cells were generated as described previously [17]. All experimental protocols were reviewed and approved by the Akita University Institutional Committee for Animal Studies. WT and KO ES cells were transfected with the GFP-ML1Nx2 expression vector using Lipofectamine2000. After 24 hrs, the cells were plated on glass-base culture dishes that had been coated with 15 μg/ml fibronectin (Sigma). Fluorescent images of live cells were acquired by a Leica AF6000 microscope.

## Results and Discussion

### Broad Endosomal Distribution of GFP-ML1Nx2

**Fig 1:**
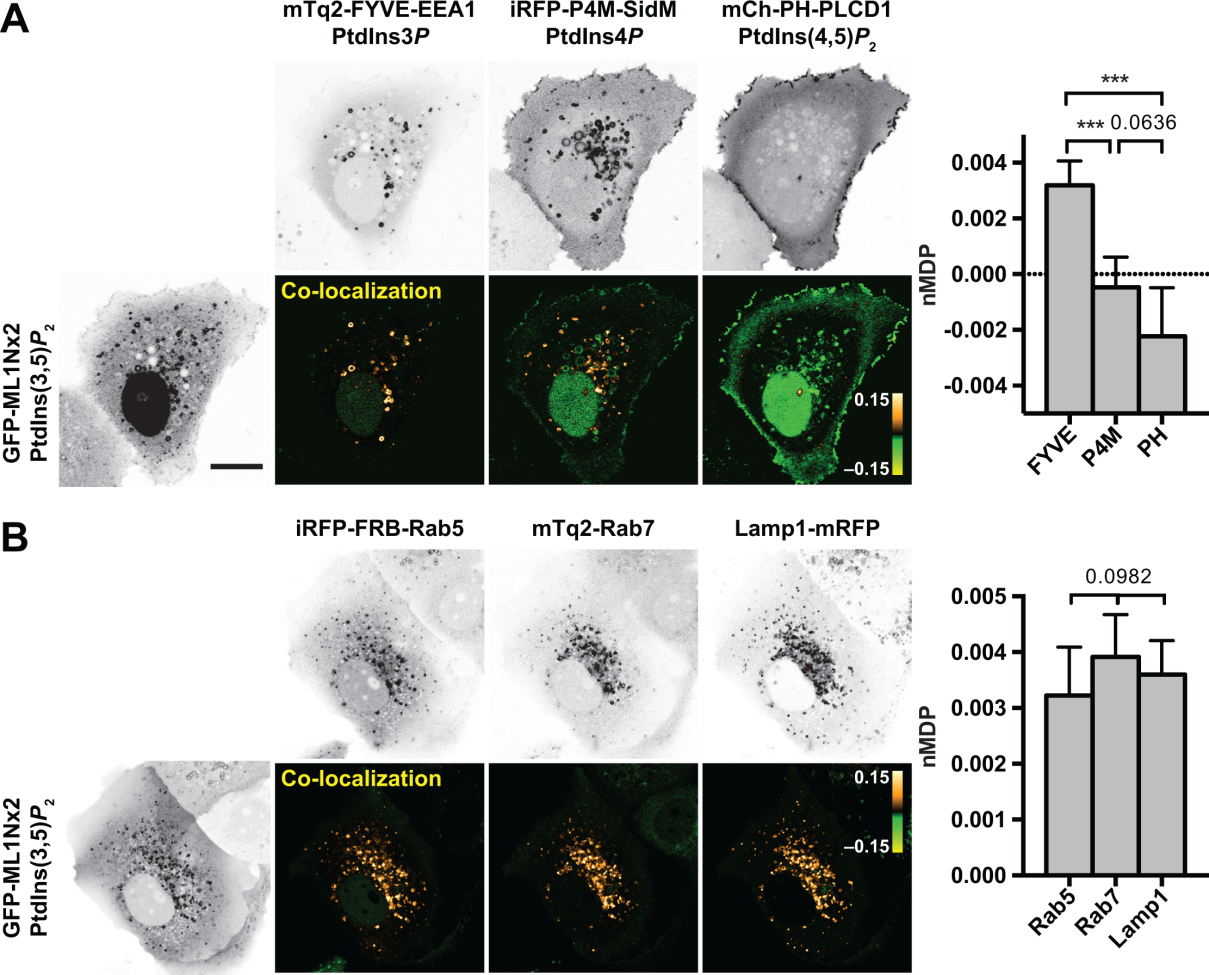
The GFP-ML1Nx2 probe labels endosomal compartments. (**A**) GFP-ML1Nx2 co-localizes more with PtdIns3*P* than other constitutive inositol lipids; the “co-localization” images show the images of the normalized mean deviation product (nMDP) between two channels, wherein bright pixels that correlate in each channel appear gold and pixels that anti-correlate appear green (dim pixels that correlate in each channel are shaded black for clarity). The graph at right shows the mean nMDP score ± 95% C.I. for 30 cells, with the *P* values represented above (One-way ANOVA with Tukey’s multiple comparison; *** = *P* < 0.0001). (**B**) GFP-ML1Nx2 co-localizes to a similar extent with early (Rab5), and late endo/lysosomal (Rab7 and LAMP1) markers. “Co-localization” images as in A; graph at right shows the mean nMDP score ± 95% C.I. for 30 cells, with the *P* values represented above (One-way ANOVA, no significant variance between groups). Scale bar = 15 μm and applies to A and B.

When expressed in COS-7 cells (Fig 1), GFP-ML1Nx2 displayed extensive overlap with mTq2-FYVE-EEA1 [34], a PtdIns3*P* marker (Fig 1A), consistent with previous observations made with ML1Nx2 [26] and as predicted from the synthesis of PtdIns(3,5)*P*_2_ from PtdIns3*P* [2]. However, despite the majority of FYVE-EEA1-positive structures exhibiting GFP-ML1Nx2 labelling, there were numerous structures positive for the ML1Nx2 but apparently negative for the PtdIns3*P* probe **(Fig 1A** and **“Cell 22”** and **“Lipids-pooled” in S1 File**).

We have previously reported a largely exclusive localization of PtdIns3*P* probes with a probe that detects late endosomal/lysosomal PtdIns4*P*, iRFP-P4M [27]. In fact, some ML1Nx2 over-lapped with P4M-positive compartments, although many puncta were only positive for one probe or the other (**Fig 1A**). Finally, the exclusively plasma membrane-labelling PtdIns(4,5)*P*_2_-probe, PH-PLCD1-mCherry [27], showed virtually no over-lap with the GFP-ML1Nx2 probe at all (**Fig 1A**). Because almost all FYVE-EEA1-positive structures were positive for ML1Nx2, but only a minority of P4M-labelled structures were positive, the highest degree of co-localization was observed with the PtdIns3*P* marker (**Fig 1A**). Nonetheless, the relatively broad distribution across PtdIns3*P*-positive early and PtdIns4*P*-positive late endosomal compartments largely agreed with the localization of ML1Nx2 reported previously [26].

To confirm the distribution across multiple endosomal compartments, we compared the localization of GFP-ML1Nx2 with the early endosomal marker, Rab5 [35], the late endosomal marker, Rab7 [35] and the lysosomal marker, Lamp1 [36,37] as shown in **Fig 1B**, (see also **“Cell 2”** in **S1 File**). Indeed, ML1Nx2 exhibited extensive co-localization with all three markers with a similar co-localization score, despite the fact that the endo/lysosomal compartment marked by Lamp1 and Rab7 (Lamp1 vs Rab7 co-localization score nMDP = 0.005937 ± 0.000981, mean 95% C.I.) was clearly resolved from the early, Rab5-postive compartment (Lamp1 vs Rab5 co-localization nMDP = 0.004281 ± 0.000676; Rab7 vs. Rab5 nMDP = 0.004613 ± 0.001115; *P* < 0.033 compared to Rab7 vs lamp1 by one-way ANOVA with Tukey’s multiple comparison, see **“Rabs-pooled”** in **S1 File**). Together, these data demonstrate ML1Nx2’s wide distribution within the endosomal system.

### Lack of PtdIns(3,5)P_2_-dependence of ML1Nx2 localization

Such a wide distribution of the ML1Nx2 reporter suggests multiple host compartments for PtdIns(3,5)*P*_2_. However, when working with fluorescent lipid biosensors, it is important to rule out the presence of accessory molecular interactions that can bias or even occlude the apparent localization of the lipid [23]. Although GFP-ML1Nx2 binding was shown to be specific for PtdIns(3,5)*P*_2_ *in vitro* [26], binding of the full-length protein was less so [25]; furthermore, *in vitro* specificity amongst lipids does not preclude other molecular interactions being necessary for membrane binding in cells. We therefore sought to confirm the specificity of the ML1Nx2 probe’s interaction with PtdIns(3,5)*P*_2_ in living cells.

**Fig 2:**
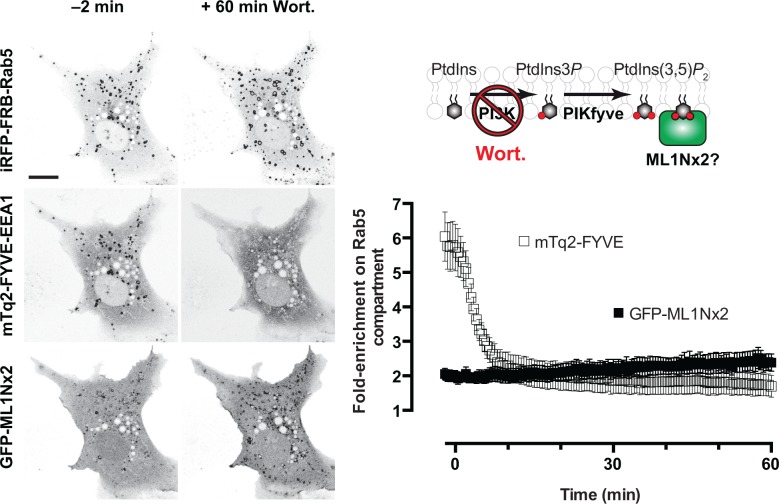
The GFP-ML1Nx2 probe does not dissociate from the Rab5-positive membranes in response to blockade of PtdIns3*P* synthesis. Images show a representative cell expressing the three indicated constructs before and 1 h after treatment with 100 nM wortmannin, which inhibits the PI 3-kinase that synthesizes PtdIns3*P*, the substrate for PtdIns(3,5)*P*_2_ synthesis. The graph at right shows mean fluorescence intensity at Rab5-positive membranes normalized to the whole cell for the indicated construct (data are means ± s.e.m. of 12 cells from three independent experiments). No dissociation of the GFP-ML1Nx2 is observed despite robust depletion of FYVE-EEA1 within 15 min of wortmannin application. Scale bar = 15 μm.

Given the sole route of synthesis of PtdIns(3,5)*P*_2_ is via 5-phosphorylation of PtdIns3*P* [5], inhibition of PtdIns3*P* synthesis would be expected to cause depletion of PtdIns(3,5)*P*_2_ (**Fig 2**). To this end, we used the broad-spectrum PI 3-kinase inhibitor wortmannin [38] at a concentration of 100 nM, which is known to effectively inhibit the PI 3-kinase Vps34 responsible for most PtdIns3*P* synthesis [39]. Time-lapse imaging revealed effective depletion of PtdIns3*P* within 10 minutes of wortmannin addition, using the FYVE-EEA1 probe, and a concomitant swelling of the Rab5-positive compartment, which stems from the stalling of PI 3-kinase-dependent trafficking at the early endosome [40,41]. Yet, no depletion of GFP-ML1Nx2 was apparent over a whole hour (**Fig 2**; see also **“Wm”** and **“Wm-pooled”** in **S2 File**). In fact, quantification of the fluorescence associated with the Rab5-positive membranes revealed a slight increase in fluorescence over 60 min, despite robust depletion of FYVE-EEA1 (**Fig 2**).

Whereas manoeuvres that inhibit PtdIns3*P* synthesis have been demonstrated to inhibit PtdIns(3,5)*P*_2_ accumulation in cells, depletion is not complete [42], potentially explaining the unyielding localization of ML1Nx2 after wortmannin addition. Indeed, given the role of PtdIns3*P* in localizing PIKfyve [42] and the fact that the PtdIns(3,5)*P*_2_-hydrolyzing Sac3 phosphatase is in complex with this kinase [12], it is easy to envision a scenario where PtdIns3*P* depletion could actually inhibit PtdIns(3,5)*P*_2_ hydrolysis. We therefore sought a more direct method to deplete this lipid in cells.

**Fig 3:**
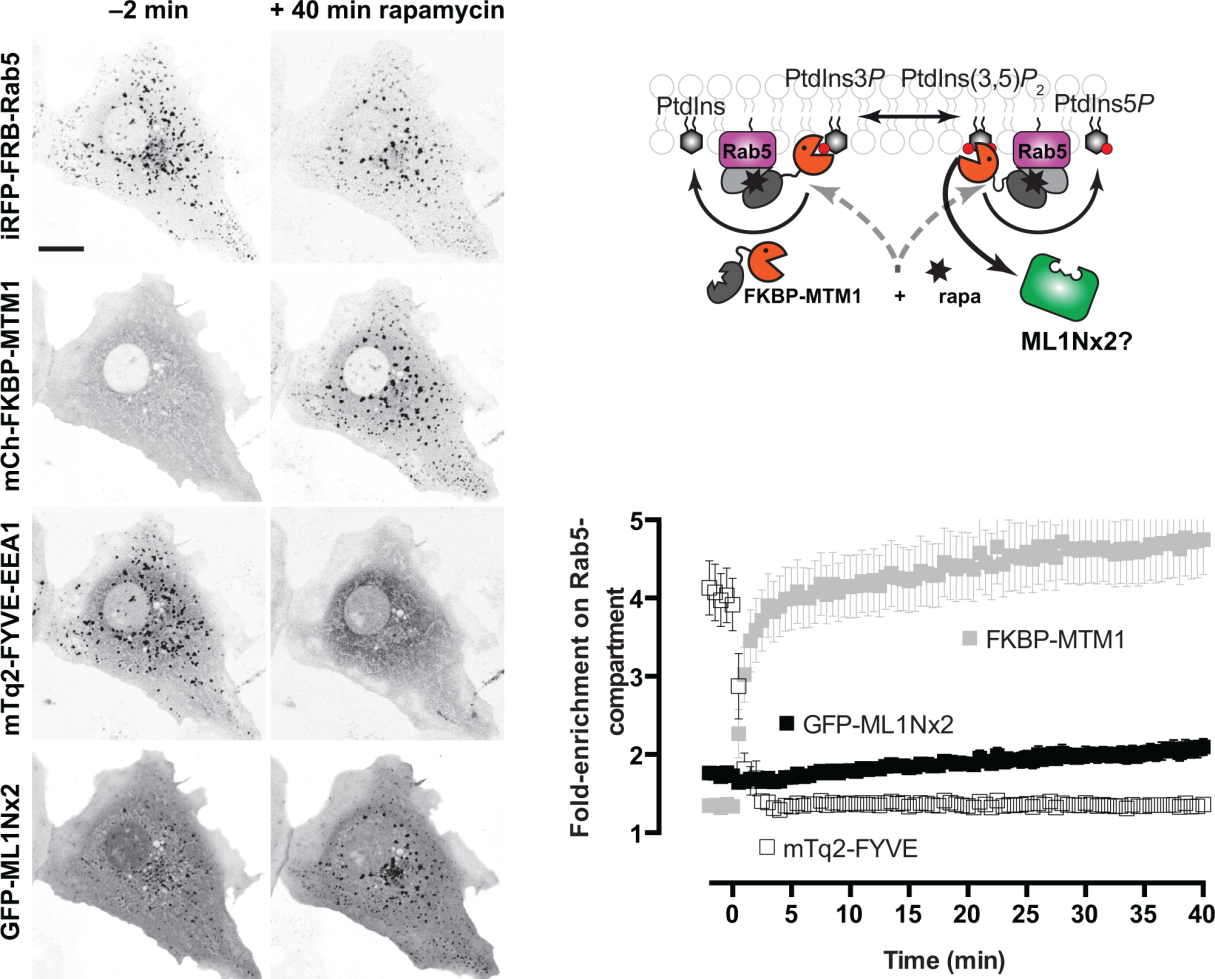
The GFP-ML1Nx2 probe does not dissociate from the Rab5-positive membranes in response to depletion of PtdIns3*P* and PdIns(3,5)*P*_2_. Images show a representative cell expressing the three indicated constructs before and 40 min after treatment with 1 μM rapamycin, which induces recruitment of FKBP-MTM1 to the FRB-Rab5-decorated membranes, thereby depleting PtdIns3*P* and PtdIns(3,5)*P*_2_. The graph at right shows mean fluorescence intensity at Rab5-positive membranes normalized to the whole cell for the indicated construct (data are means ± s.e.m. of 15 cells from four independent experiments). No dissociation of the GFP-ML1Nx2 is observed despite robust depletion of FYVE-EEA1 within 2 min of rapamycin application. Scale bar = 15 μm.

The myotubularin phosphatase MTM1 is known to hydrolyze the 3-phosphate from both PtdIns3*P* and PtdIns(3,5)*P*_2_ [13]. The enzyme was previously used in conjunction with rapamycin-induced chemical dimerization of FKBP and FRB-fused proteins to acutely recruit it to Rab5-positive membranes and acutely deplete the lipids [43]. We utilized this strategy to test the specificity of ML1Nx2 (**Fig 3**). Rapamycin induced robust recruitment of an FKBP-conjugated MTM1 enzyme to Rab5-positive membranes, and rapid and complete elimination of FYVE-EEA1 labelling in this compartment–with no decrease in GFP-ML1Nx2 labelling (**Fig 3**; see also **“MTM”** and **“BMTM-pooled”** in **S3 File**). In fact, as with wortmannin treatment, a slight increase in labelling was observed. These observations are inconsistent with GFP-ML1Nx2 localizing to Rab5-positive membranes in a PtdIns(3,5)*P*_2_-dependent and specific manner.

**Fig 4:**
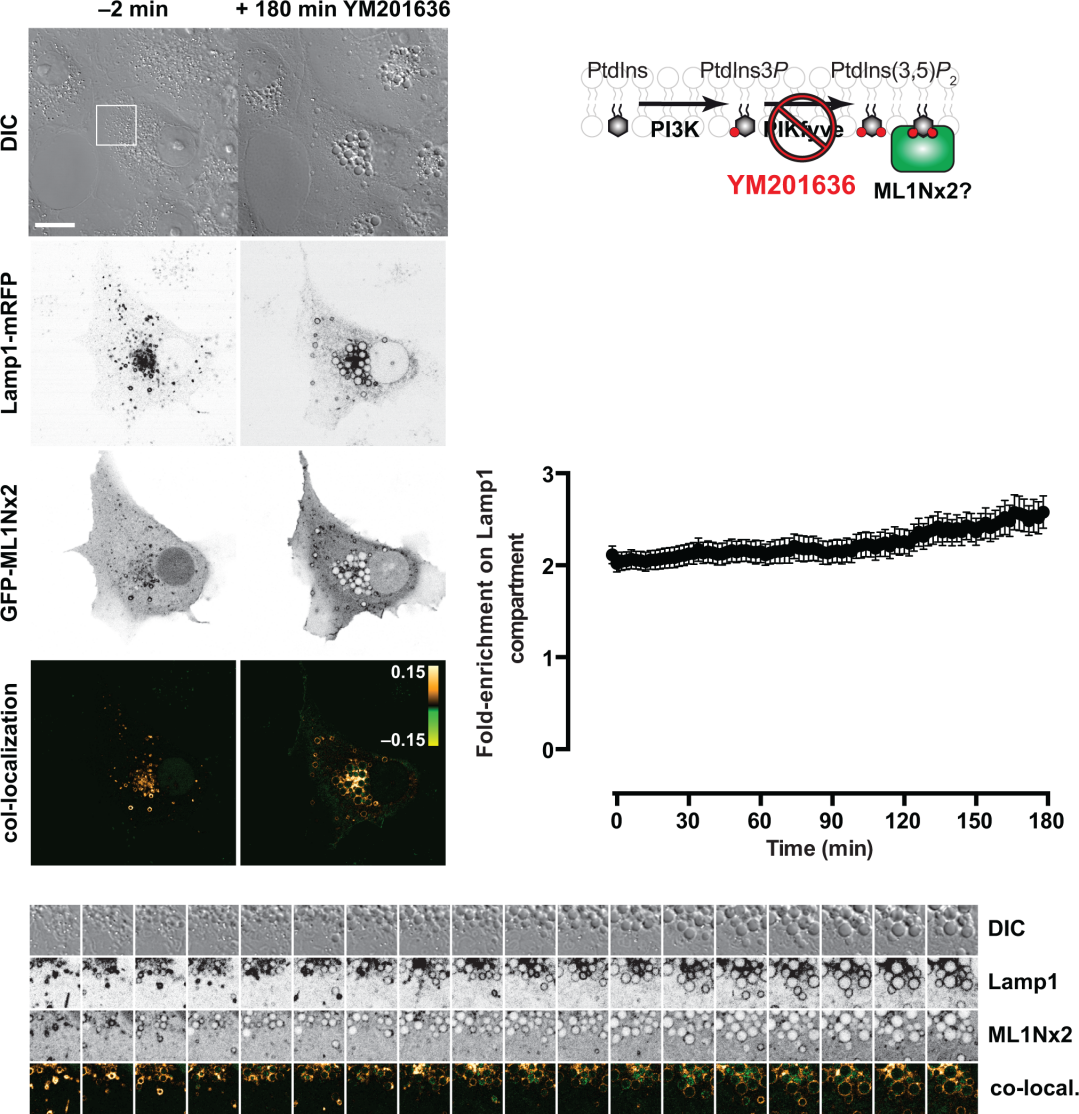
The GFP-ML1Nx2 probe does not dissociate from the LAMP1-positive membranes in response to blockade of PtdIns(3,5)*P*_2_ synthesis. Images show a representative cell expressing the indicated constructs before and 3 h after treatment with 1 μM YM201636, which inhibits PIKfyve that synthesizes PtdIns(3,5)*P*_2_. The montage images at the bottom are from the boxed region above, and are separated by 10 min intervals. The graph at right shows mean fluorescence intensity at LAMP1-positive membranes normalized to the whole cell for the indicated construct (data are means ± s.e.m. of 29 cells from three independent experiments). No dissociation of the GFP-ML1Nx2 is observed despite extensive vacuolation of the cells in response to YM201636 treatment. Scale bar = 15 μm.

Previous experiments with GFP-ML1Nx2 demonstrated cellular specificity of the probe through pharmacological inhibition of PIKfyve [26] with YM201636, a compound known to produce rapid depletion of the lipid [16,44]. We sought to repeat these experiments via time-lapse imaging, to follow the effect of the compound on living cells (**Fig 4** and accompanying raw **tiff** files in **S4 File**). The compound was effective, producing the characteristic swollen vacuole phenotype that results from PtdIns(3,5)*P*_2_ inhibition [16]–yet we observed no global decreases in GFP-ML1Nx2 labelling. Quantitative analysis of fluorescence intensity with a Lamp1-mRFP marker again revealed only a slight increase in overall binding at this compartment (**Fig 4**; see also **“pooled”** in **S4 File**). Because the individual vesicular structures labelled inside cells are motile, continuously splitting, merging and moving out of the plane of focus, it is not possible to track the association of GFP-ML1Nx2 with individual structures with any confidence. None the less, the data clearly show no overall decrease in GFP-ML1Nx2 association with Lamp1-positive membranes after elimination of PtdIns(3,5)*P*_2_ with YM201636.

These results are in contrast to those reported previously [26]. However, we believe our data are a more rigorous assessment as they were achieved by time-lapse imaging, whereas the previous study was a cohort approach, comparing separate cell populations treated with YM201636 or vehicle. Therefore, variations between cells in terms of expression level and morphology may have accounted for the differences observed, rather than as a direct result of PtdIns(3,5)*P*_2_ elimination. Notably, a maximal effect was only achieved after 24 hours in the previous study [26], far longer than the matter of 1–2 hours for vacuolation to develop (**Fig 4**) and the few minutes necessary for PtdIns(3,5)*P*_2_-depletion [16,44]; this argues strongly for an indirect effect as the cause of decreased GFP-ML1Nx2 localization in the previous study.

**Fig 5:**
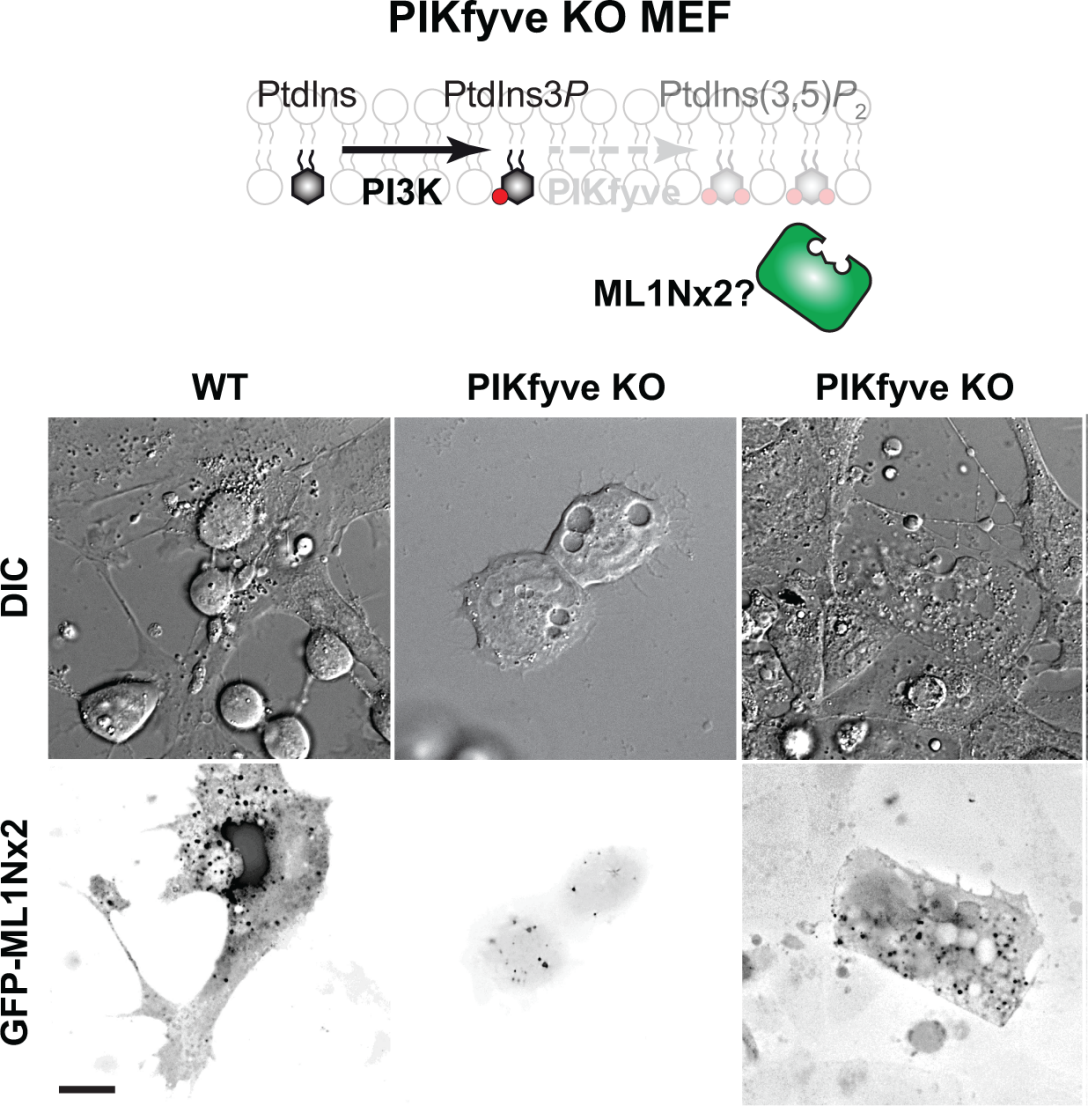
The GFP-ML1Nx2 probe labels punctate structures even in the absence of PIKfyve. Images show a representative wild-type and PIKfyve knock-out (KO) ES cells expressing the GFP-ML1Nx2 probe. Note the fluorescent puncta in the PIKfyve KO cell which showed swollen endosomes, a hallmark of PtdIns(3,5)*P*_2_ deficiency. Data are representative of 11 cells, scale bar = 15 μm.

As a final test of the PtdIns(3,5)*P*_2_-dependece of GFP-ML1Nx2 localization in cells, we turned to our extensively characterized murine embryonic fibroblasts (MEF) null for PIKfyve, which are unable to synthesize the lipid [17]. These cells exhibit the swollen vacuoles in their cytoplasm (**Fig 5**; see accompanying **“jpeg”** files in **S5 File**), yet still exhibit clear vesicular distribution of GFP-ML1Nx2. Therefore, some cellular component evidently enables GFP-ML1Nx2 to localize to membranes in the absence of PtdIns(3,5)*P*_2_.

## Conclusions

Having applied a battery of pharmacological, enzymatic and genetic approaches to deplete PtdIns(3,5)*P*_2_ from cells, we conclude that the continued localization of the GFP-ML1Nx2 biosensor to endosomal membranes can only be due to its interaction with some cellular component other than PtdIns(3,5)*P*_2_. What this other component may be is not indicated by our data, and is not strictly relevant to our study; what is clear is that PtdIns(3,5)*P*_2_ is not necessary for membrane localization of GFP-ML1Nx2 in cells. Given the in vitro interaction of this protein with the lipid [25,26], it is perhaps possible that PtdIns(3,5)*P*_2_ is sufficient to localize the probe to some subset of cellular structures, and therefore these structures are truly labelled due to the presence of the lipid. However, the alternative interaction, whatever it may be, occludes any specificity in the cellular context; it is not possible to identify whether a GFP-ML1Nx2-labelled compartment is labelled due to the presence of PtdIns(3,5)*P*_2_ *a priori*. We therefore urge caution in interpreting the localization of GFP-ML1Nx2 as a biosensor for the localization or relative abundance of PtdIns(3,5)*P*_2_ in cells.

## Supporting Information

S1 File Raw data accompanying Fig 1. The folder labeled “Cell 2” contains raw images as well as the nMDP images of the cell shown in Fig 1A. “Lipids-pooled” is the Graphpad Prism spreadsheet containing nMDP data from all cells analyzed and plotted in the bar graph in Fig 1A. The folder labeled “Cell 22” contains raw images as well as the nMDP images of the cell shown in Fig 1B. “Rabs-pooled” is the Graphpad Prism spreadsheet containing nMDP data from all cells analyzed and plotted in the bar graph in Fig 1A.
(ZIP)

S2 File Raw data accompanying Fig 2. The folder labeled “Wm” contains raw images of the cell shown in Fig 2. “Wm-pooled” is the Graphpad Prism spreadsheet containing normalized intensity data from all cells analyzed and plotted in the graph in Fig 2.
(ZIP)

S3 File Raw data accompanying Fig 3. The folder labeled “MTM” contains raw images of the cell shown in Fig 3. “MTM-pooled” is the Graphpad Prism spreadsheet containing normalized intensity data from all cells analyzed and plotted in the graph in Fig 3.
(ZIP)

S4 File Raw data accompanying Fig 4. Raw “tiff” image files of the cell shown in Fig 4. “pooled” is the Graphpad Prism spreadsheet containing normalized intensity and nMDP data from all cells analyzed and plotted in the graph in Fig 4.
(ZIP)

S5 File Raw data accompanying Fig 5. Raw “jpeg” image files of the cell shown in Fig 4.
(ZIP)
