# Supplementary figures and images for: The ML1Nx2 Phosphatidylinositol 3,5-Bisphosphate Probe Shows Poor Selectivity in Cells
Source: PLoS One. 2015 Oct 13;10(10):e0139957. doi: 10.1371/journal.pone.0139957 (PMC4604148; doi:10.1371/journal.pone.0139957)

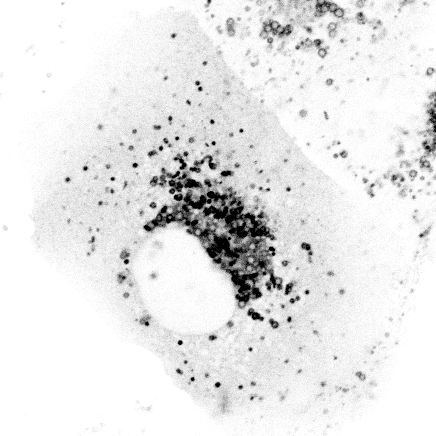

Supplement: S1 File — The folder labeled “Cell 2” contains raw images as well as the nMDP images of the cell shown in Fig 1A. “Lipids-pooled” is the Graphpad Prism spreadsheet containing nMDP data from all cells analyzed and plotted in the bar graph in Fig 1A. The folder labeled “Cell 22” contains raw images as well as the nMDP images of the cell shown in Fig 1B. “Rabs-pooled” is the Graphpad Prism spreadsheet containing nMDP data from all cells analyzed and plotted in the bar graph in Fig 1A. (ZIP) [file pone.0139957.s001.zip › Fig1/Cell2/Lamp1-2.tif]

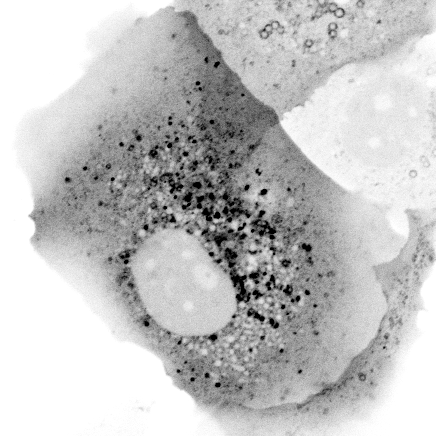

Supplement: S1 File — The folder labeled “Cell 2” contains raw images as well as the nMDP images of the cell shown in Fig 1A. “Lipids-pooled” is the Graphpad Prism spreadsheet containing nMDP data from all cells analyzed and plotted in the bar graph in Fig 1A. The folder labeled “Cell 22” contains raw images as well as the nMDP images of the cell shown in Fig 1B. “Rabs-pooled” is the Graphpad Prism spreadsheet containing nMDP data from all cells analyzed and plotted in the bar graph in Fig 1A. (ZIP) [file pone.0139957.s001.zip › Fig1/Cell2/ML1Nx2-2.tif]

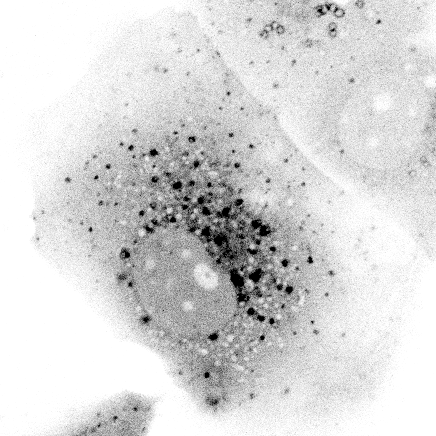

Supplement: S1 File — The folder labeled “Cell 2” contains raw images as well as the nMDP images of the cell shown in Fig 1A. “Lipids-pooled” is the Graphpad Prism spreadsheet containing nMDP data from all cells analyzed and plotted in the bar graph in Fig 1A. The folder labeled “Cell 22” contains raw images as well as the nMDP images of the cell shown in Fig 1B. “Rabs-pooled” is the Graphpad Prism spreadsheet containing nMDP data from all cells analyzed and plotted in the bar graph in Fig 1A. (ZIP) [file pone.0139957.s001.zip › Fig1/Cell2/Rab5-2.tif]

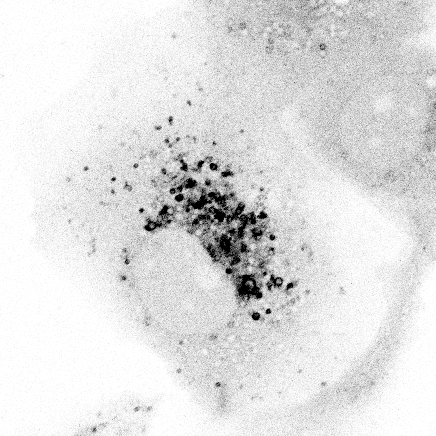

Supplement: S1 File — The folder labeled “Cell 2” contains raw images as well as the nMDP images of the cell shown in Fig 1A. “Lipids-pooled” is the Graphpad Prism spreadsheet containing nMDP data from all cells analyzed and plotted in the bar graph in Fig 1A. The folder labeled “Cell 22” contains raw images as well as the nMDP images of the cell shown in Fig 1B. “Rabs-pooled” is the Graphpad Prism spreadsheet containing nMDP data from all cells analyzed and plotted in the bar graph in Fig 1A. (ZIP) [file pone.0139957.s001.zip › Fig1/Cell2/Rab7-2.tif]

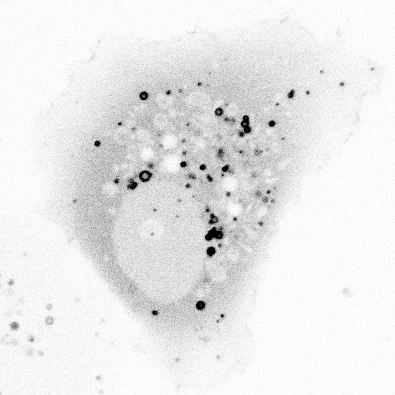

Supplement: S1 File — The folder labeled “Cell 2” contains raw images as well as the nMDP images of the cell shown in Fig 1A. “Lipids-pooled” is the Graphpad Prism spreadsheet containing nMDP data from all cells analyzed and plotted in the bar graph in Fig 1A. The folder labeled “Cell 22” contains raw images as well as the nMDP images of the cell shown in Fig 1B. “Rabs-pooled” is the Graphpad Prism spreadsheet containing nMDP data from all cells analyzed and plotted in the bar graph in Fig 1A. (ZIP) [file pone.0139957.s001.zip › Fig1/Cell22/FYVE-22.tif]

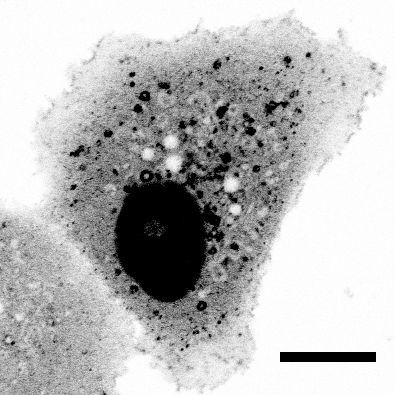

Supplement: S1 File — The folder labeled “Cell 2” contains raw images as well as the nMDP images of the cell shown in Fig 1A. “Lipids-pooled” is the Graphpad Prism spreadsheet containing nMDP data from all cells analyzed and plotted in the bar graph in Fig 1A. The folder labeled “Cell 22” contains raw images as well as the nMDP images of the cell shown in Fig 1B. “Rabs-pooled” is the Graphpad Prism spreadsheet containing nMDP data from all cells analyzed and plotted in the bar graph in Fig 1A. (ZIP) [file pone.0139957.s001.zip › Fig1/Cell22/ML1Nx2-22-15um.tif]

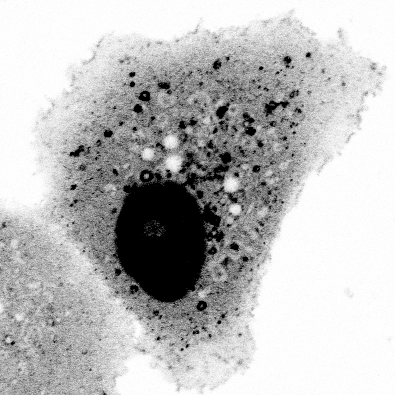

Supplement: S1 File — The folder labeled “Cell 2” contains raw images as well as the nMDP images of the cell shown in Fig 1A. “Lipids-pooled” is the Graphpad Prism spreadsheet containing nMDP data from all cells analyzed and plotted in the bar graph in Fig 1A. The folder labeled “Cell 22” contains raw images as well as the nMDP images of the cell shown in Fig 1B. “Rabs-pooled” is the Graphpad Prism spreadsheet containing nMDP data from all cells analyzed and plotted in the bar graph in Fig 1A. (ZIP) [file pone.0139957.s001.zip › Fig1/Cell22/ML1Nx2-22.tif]

nMDP

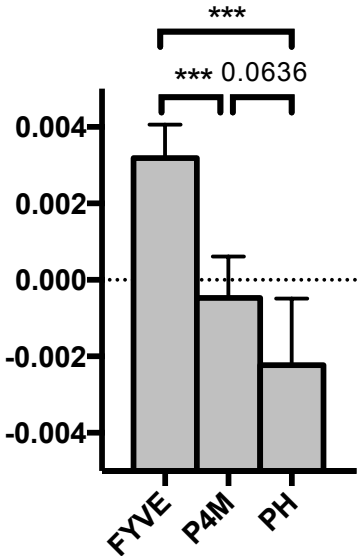

Supplement: S1 File — The folder labeled “Cell 2” contains raw images as well as the nMDP images of the cell shown in Fig 1A. “Lipids-pooled” is the Graphpad Prism spreadsheet containing nMDP data from all cells analyzed and plotted in the bar graph in Fig 1A. The folder labeled “Cell 22” contains raw images as well as the nMDP images of the cell shown in Fig 1B. “Rabs-pooled” is the Graphpad Prism spreadsheet containing nMDP data from all cells analyzed and plotted in the bar graph in Fig 1A. (ZIP) [file pone.0139957.s001.zip › Fig1/Cell22/nMDP.pdf]

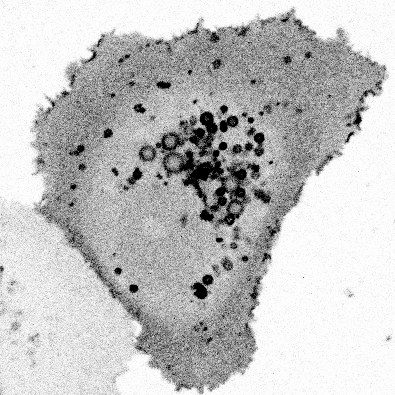

Supplement: S1 File — The folder labeled “Cell 2” contains raw images as well as the nMDP images of the cell shown in Fig 1A. “Lipids-pooled” is the Graphpad Prism spreadsheet containing nMDP data from all cells analyzed and plotted in the bar graph in Fig 1A. The folder labeled “Cell 22” contains raw images as well as the nMDP images of the cell shown in Fig 1B. “Rabs-pooled” is the Graphpad Prism spreadsheet containing nMDP data from all cells analyzed and plotted in the bar graph in Fig 1A. (ZIP) [file pone.0139957.s001.zip › Fig1/Cell22/P4M-22.tif]

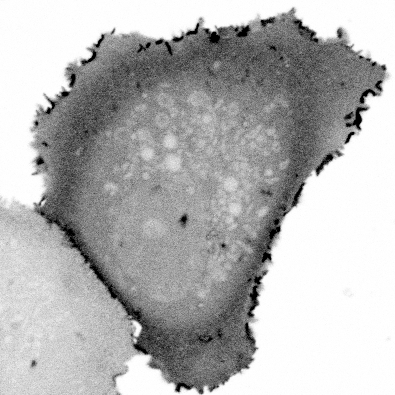

Supplement: S1 File — The folder labeled “Cell 2” contains raw images as well as the nMDP images of the cell shown in Fig 1A. “Lipids-pooled” is the Graphpad Prism spreadsheet containing nMDP data from all cells analyzed and plotted in the bar graph in Fig 1A. The folder labeled “Cell 22” contains raw images as well as the nMDP images of the cell shown in Fig 1B. “Rabs-pooled” is the Graphpad Prism spreadsheet containing nMDP data from all cells analyzed and plotted in the bar graph in Fig 1A. (ZIP) [file pone.0139957.s001.zip › Fig1/Cell22/PH-22.tif]

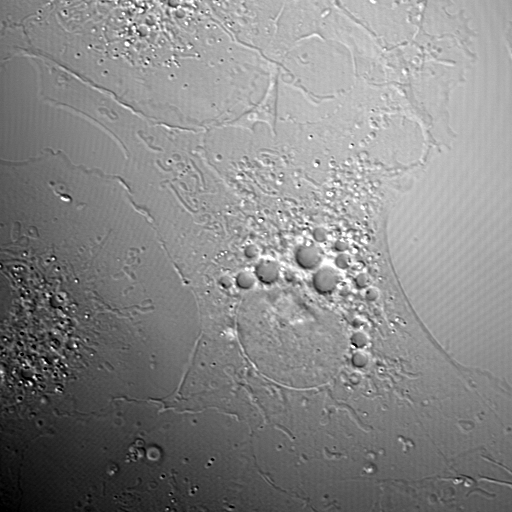

Supplement: S2 File — The folder labeled “Wm” contains raw images of the cell shown in Fig 2. “Wm-pooled” is the Graphpad Prism spreadsheet containing normalized intensity data from all cells analyzed and plotted in the graph in Fig 2. (ZIP) [file pone.0139957.s002.zip › Fig2/Wm/DIC-1.tif]

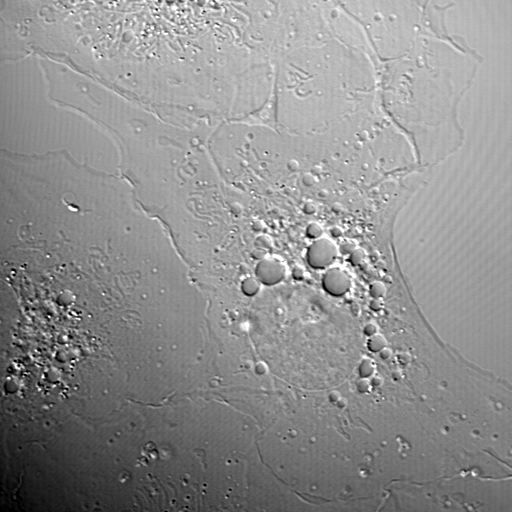

Supplement: S2 File — The folder labeled “Wm” contains raw images of the cell shown in Fig 2. “Wm-pooled” is the Graphpad Prism spreadsheet containing normalized intensity data from all cells analyzed and plotted in the graph in Fig 2. (ZIP) [file pone.0139957.s002.zip › Fig2/Wm/DIC-2.tif]

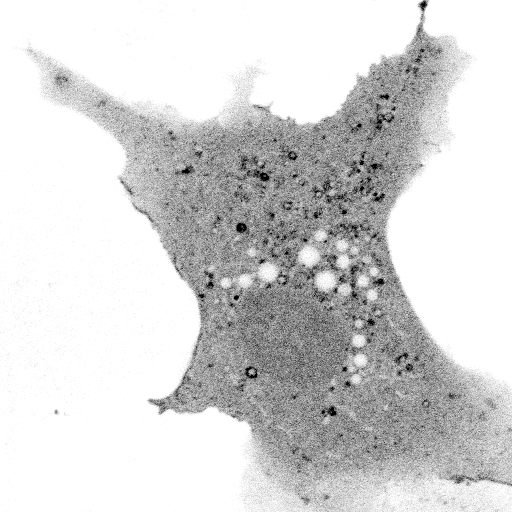

Supplement: S2 File — The folder labeled “Wm” contains raw images of the cell shown in Fig 2. “Wm-pooled” is the Graphpad Prism spreadsheet containing normalized intensity data from all cells analyzed and plotted in the graph in Fig 2. (ZIP) [file pone.0139957.s002.zip › Fig2/Wm/ML1Nx2-1.tif]

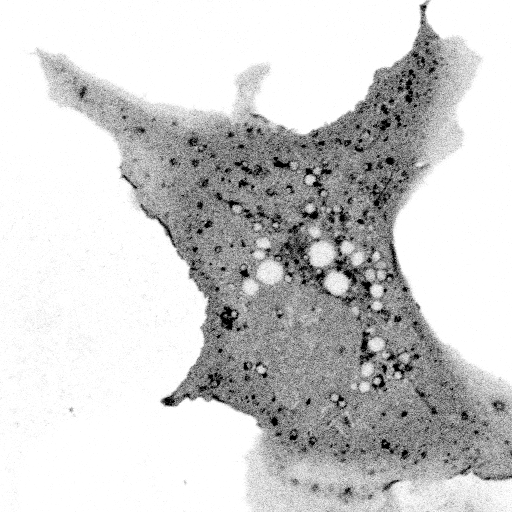

Supplement: S2 File — The folder labeled “Wm” contains raw images of the cell shown in Fig 2. “Wm-pooled” is the Graphpad Prism spreadsheet containing normalized intensity data from all cells analyzed and plotted in the graph in Fig 2. (ZIP) [file pone.0139957.s002.zip › Fig2/Wm/ML1Nx2-2.tif]

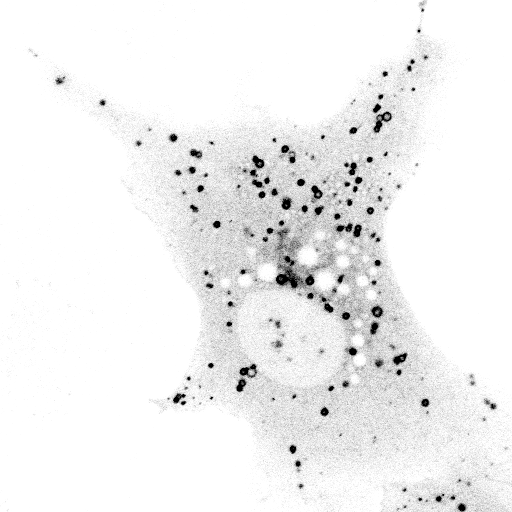

Supplement: S2 File — The folder labeled “Wm” contains raw images of the cell shown in Fig 2. “Wm-pooled” is the Graphpad Prism spreadsheet containing normalized intensity data from all cells analyzed and plotted in the graph in Fig 2. (ZIP) [file pone.0139957.s002.zip › Fig2/Wm/Rab5-1.tif]

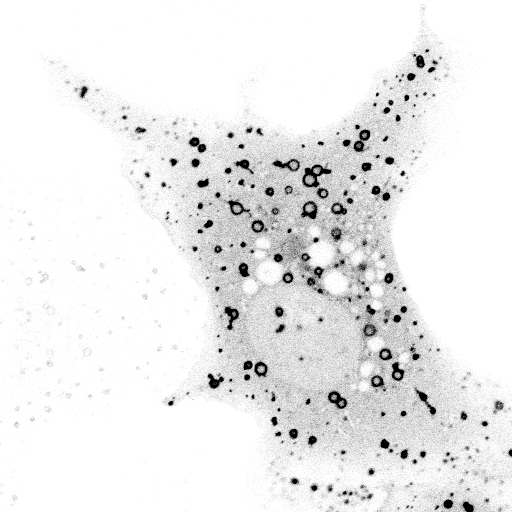

Supplement: S2 File — The folder labeled “Wm” contains raw images of the cell shown in Fig 2. “Wm-pooled” is the Graphpad Prism spreadsheet containing normalized intensity data from all cells analyzed and plotted in the graph in Fig 2. (ZIP) [file pone.0139957.s002.zip › Fig2/Wm/Rab5-2.tif]

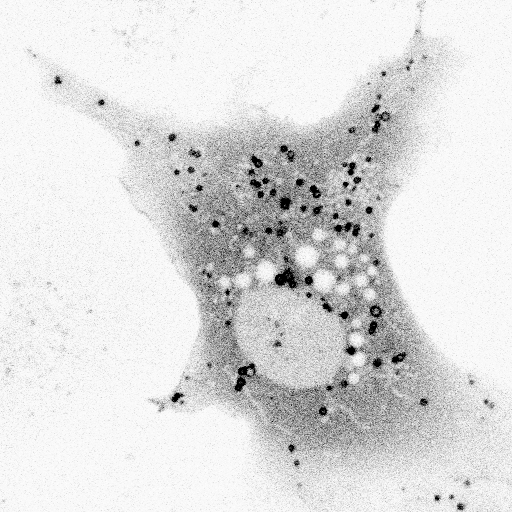

Supplement: S2 File — The folder labeled “Wm” contains raw images of the cell shown in Fig 2. “Wm-pooled” is the Graphpad Prism spreadsheet containing normalized intensity data from all cells analyzed and plotted in the graph in Fig 2. (ZIP) [file pone.0139957.s002.zip › Fig2/Wm/Wm-1.tif]

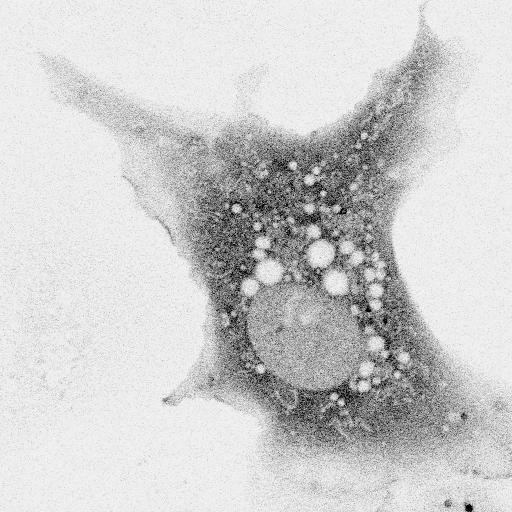

Supplement: S2 File — The folder labeled “Wm” contains raw images of the cell shown in Fig 2. “Wm-pooled” is the Graphpad Prism spreadsheet containing normalized intensity data from all cells analyzed and plotted in the graph in Fig 2. (ZIP) [file pone.0139957.s002.zip › Fig2/Wm/Wm-2.tif]

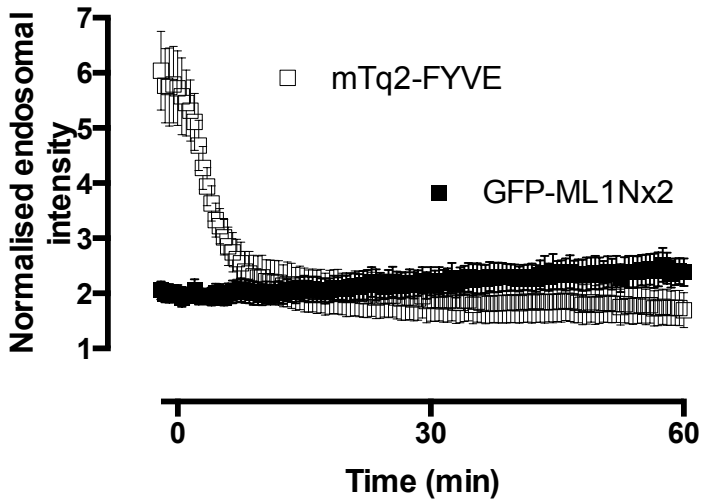

Supplement: S2 File — The folder labeled “Wm” contains raw images of the cell shown in Fig 2. “Wm-pooled” is the Graphpad Prism spreadsheet containing normalized intensity data from all cells analyzed and plotted in the graph in Fig 2. (ZIP) [file pone.0139957.s002.zip › Fig2/Wm/Wm-pooled.pdf]

Relative intensity at Rab5

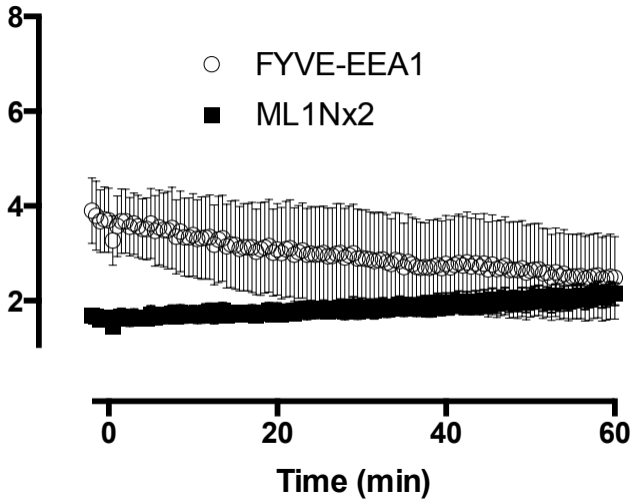

Supplement: S2 File — The folder labeled “Wm” contains raw images of the cell shown in Fig 2. “Wm-pooled” is the Graphpad Prism spreadsheet containing normalized intensity data from all cells analyzed and plotted in the graph in Fig 2. (ZIP) [file pone.0139957.s002.zip › Fig2/Wm/Wm.pdf]

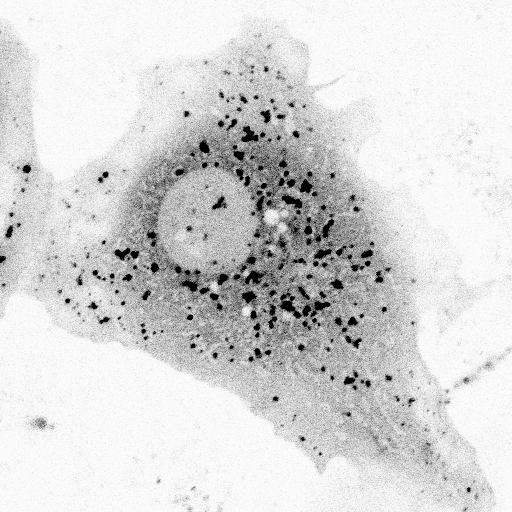

Supplement: S3 File — The folder labeled “MTM” contains raw images of the cell shown in Fig 3. “MTM-pooled” is the Graphpad Prism spreadsheet containing normalized intensity data from all cells analyzed and plotted in the graph in Fig 3. (ZIP) [file pone.0139957.s003.zip › Fig3/MTM/FYVE-1.tif]

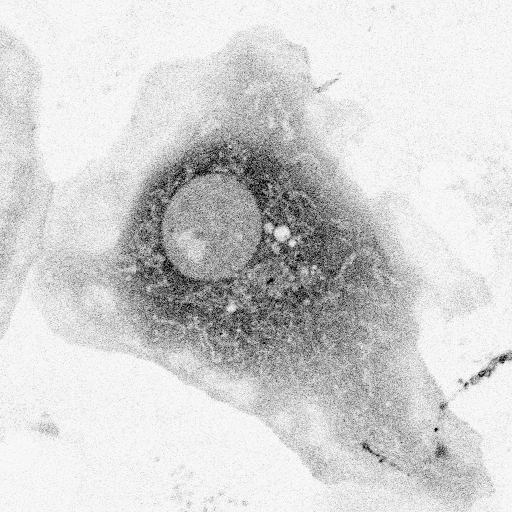

Supplement: S3 File — The folder labeled “MTM” contains raw images of the cell shown in Fig 3. “MTM-pooled” is the Graphpad Prism spreadsheet containing normalized intensity data from all cells analyzed and plotted in the graph in Fig 3. (ZIP) [file pone.0139957.s003.zip › Fig3/MTM/FYVE-2.tif]

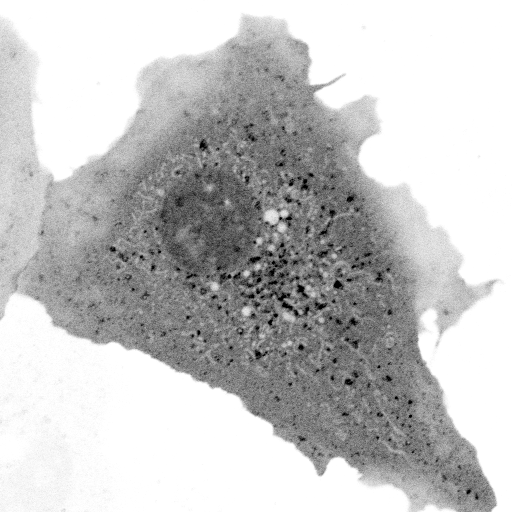

Supplement: S3 File — The folder labeled “MTM” contains raw images of the cell shown in Fig 3. “MTM-pooled” is the Graphpad Prism spreadsheet containing normalized intensity data from all cells analyzed and plotted in the graph in Fig 3. (ZIP) [file pone.0139957.s003.zip › Fig3/MTM/ML1Nx2-1.tif]

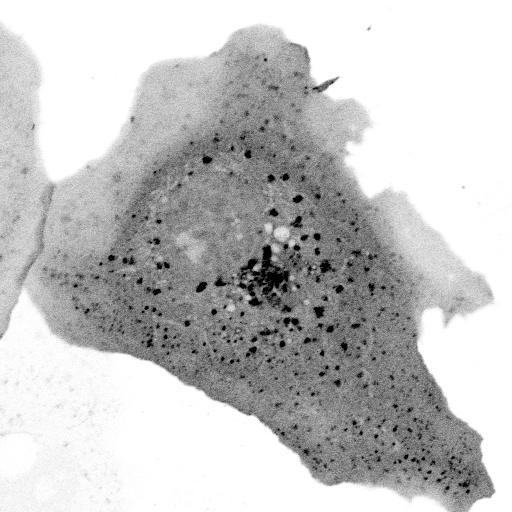

Supplement: S3 File — The folder labeled “MTM” contains raw images of the cell shown in Fig 3. “MTM-pooled” is the Graphpad Prism spreadsheet containing normalized intensity data from all cells analyzed and plotted in the graph in Fig 3. (ZIP) [file pone.0139957.s003.zip › Fig3/MTM/ML1Nx2-2.tif]

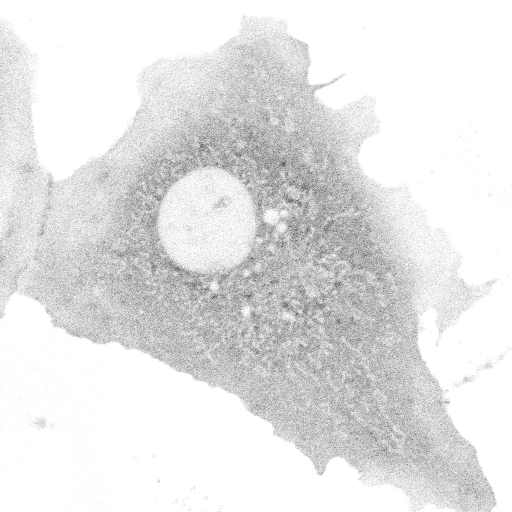

Supplement: S3 File — The folder labeled “MTM” contains raw images of the cell shown in Fig 3. “MTM-pooled” is the Graphpad Prism spreadsheet containing normalized intensity data from all cells analyzed and plotted in the graph in Fig 3. (ZIP) [file pone.0139957.s003.zip › Fig3/MTM/MTM-1.tif]

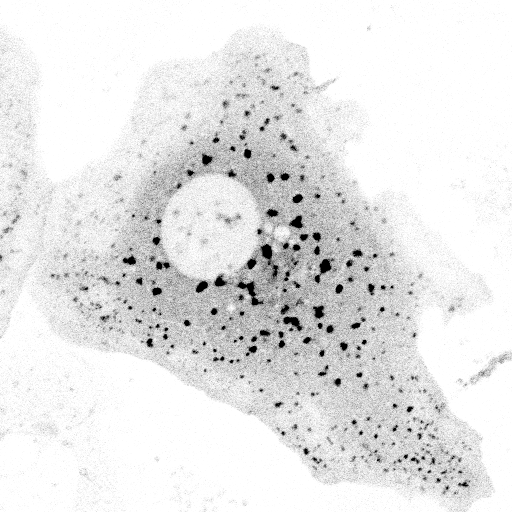

Supplement: S3 File — The folder labeled “MTM” contains raw images of the cell shown in Fig 3. “MTM-pooled” is the Graphpad Prism spreadsheet containing normalized intensity data from all cells analyzed and plotted in the graph in Fig 3. (ZIP) [file pone.0139957.s003.zip › Fig3/MTM/MTM-2.tif]

# MTM

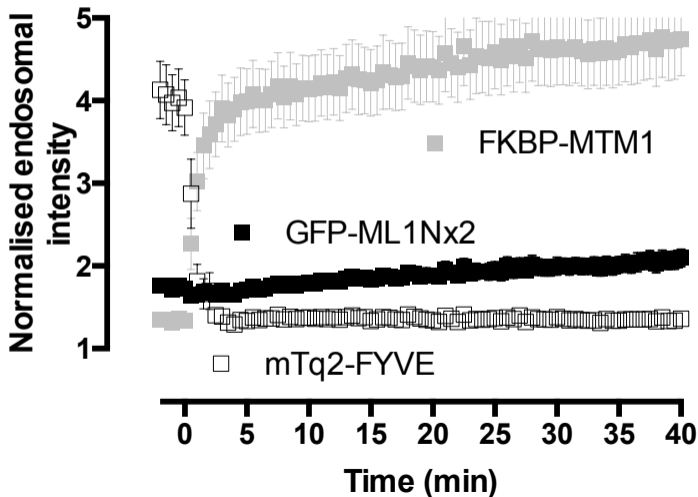

Supplement: S3 File — The folder labeled “MTM” contains raw images of the cell shown in Fig 3. “MTM-pooled” is the Graphpad Prism spreadsheet containing normalized intensity data from all cells analyzed and plotted in the graph in Fig 3. (ZIP) [file pone.0139957.s003.zip › Fig3/MTM/MTM-pooled.pdf]

Relative intensity at Rab5

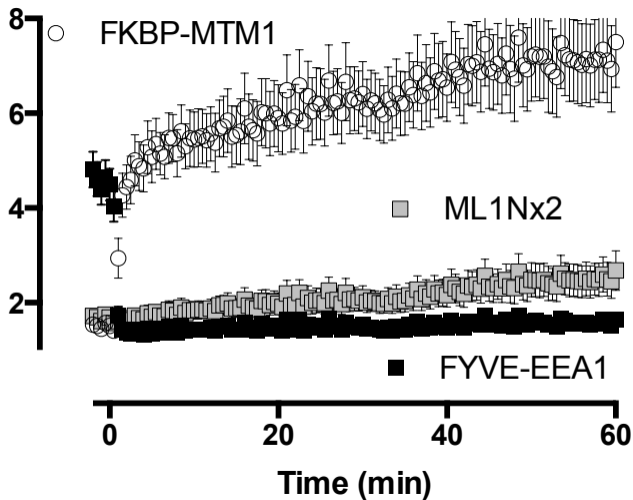

Supplement: S3 File — The folder labeled “MTM” contains raw images of the cell shown in Fig 3. “MTM-pooled” is the Graphpad Prism spreadsheet containing normalized intensity data from all cells analyzed and plotted in the graph in Fig 3. (ZIP) [file pone.0139957.s003.zip › Fig3/MTM/MTM1.pdf]

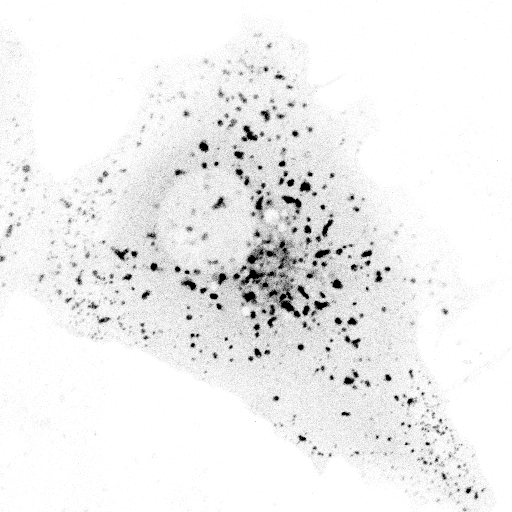

Supplement: S3 File — The folder labeled “MTM” contains raw images of the cell shown in Fig 3. “MTM-pooled” is the Graphpad Prism spreadsheet containing normalized intensity data from all cells analyzed and plotted in the graph in Fig 3. (ZIP) [file pone.0139957.s003.zip › Fig3/MTM/Rab5-1.tif]

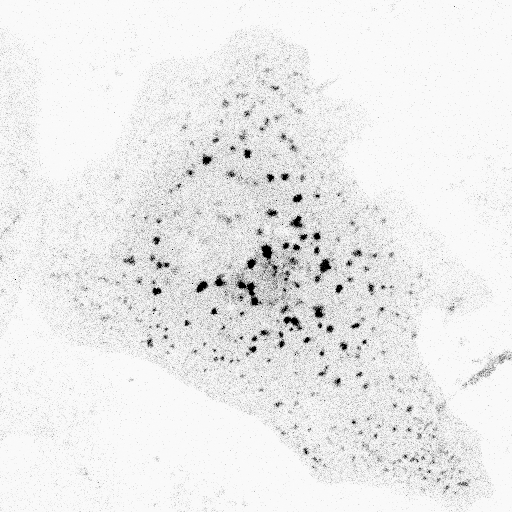

Supplement: S3 File — The folder labeled “MTM” contains raw images of the cell shown in Fig 3. “MTM-pooled” is the Graphpad Prism spreadsheet containing normalized intensity data from all cells analyzed and plotted in the graph in Fig 3. (ZIP) [file pone.0139957.s003.zip › Fig3/MTM/Rab5-2.tif]

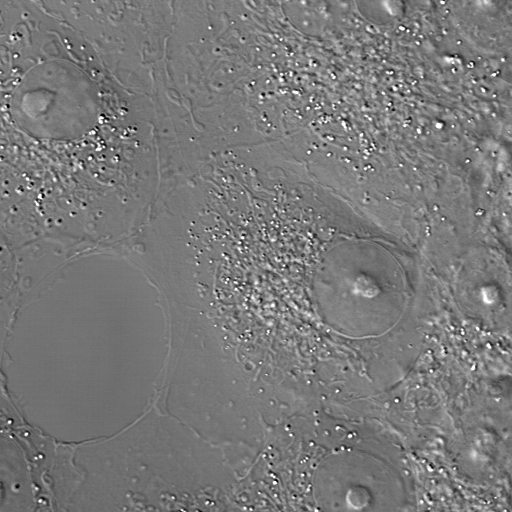

Supplement: S4 File — Raw “tiff” image files of the cell shown in Fig 4. “pooled” is the Graphpad Prism spreadsheet containing normalized intensity and nMDP data from all cells analyzed and plotted in the graph in Fig 4. (ZIP) [file pone.0139957.s004.zip › Fig4/DIC--2.tif]

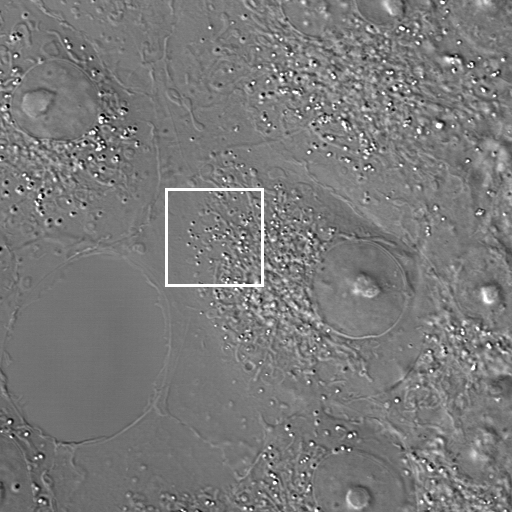

Supplement: S4 File — Raw “tiff” image files of the cell shown in Fig 4. “pooled” is the Graphpad Prism spreadsheet containing normalized intensity and nMDP data from all cells analyzed and plotted in the graph in Fig 4. (ZIP) [file pone.0139957.s004.zip › Fig4/DIC--2roi.tif]

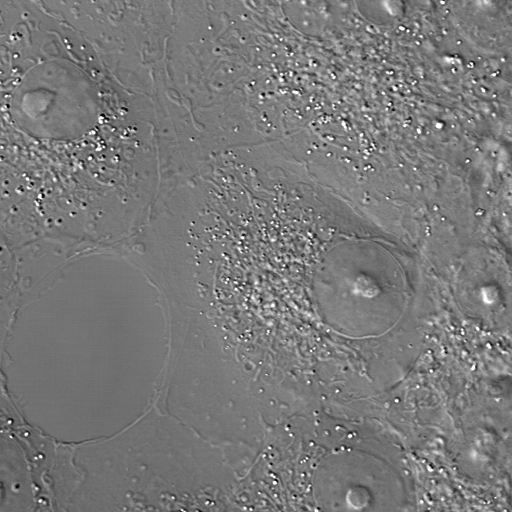

Supplement: S4 File — Raw “tiff” image files of the cell shown in Fig 4. “pooled” is the Graphpad Prism spreadsheet containing normalized intensity and nMDP data from all cells analyzed and plotted in the graph in Fig 4. (ZIP) [file pone.0139957.s004.zip › Fig4/DIC-1.tif]

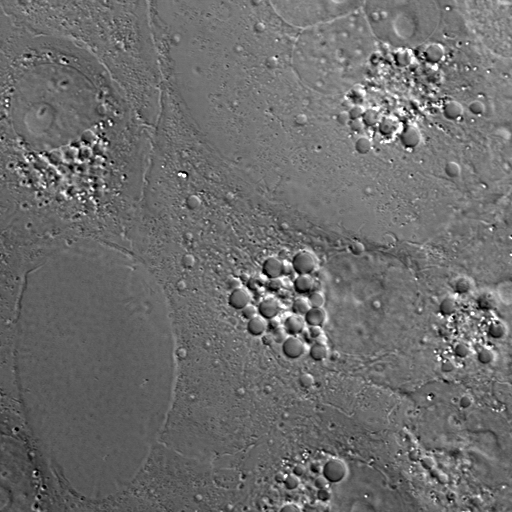

Supplement: S4 File — Raw “tiff” image files of the cell shown in Fig 4. “pooled” is the Graphpad Prism spreadsheet containing normalized intensity and nMDP data from all cells analyzed and plotted in the graph in Fig 4. (ZIP) [file pone.0139957.s004.zip › Fig4/DIC-180.tif]

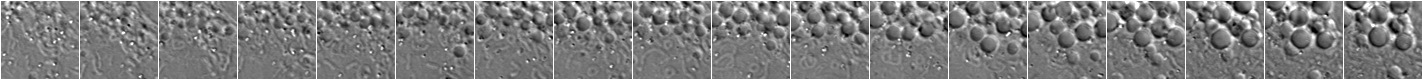

Supplement: S4 File — Raw “tiff” image files of the cell shown in Fig 4. “pooled” is the Graphpad Prism spreadsheet containing normalized intensity and nMDP data from all cells analyzed and plotted in the graph in Fig 4. (ZIP) [file pone.0139957.s004.zip › Fig4/DIC-montage.tif]

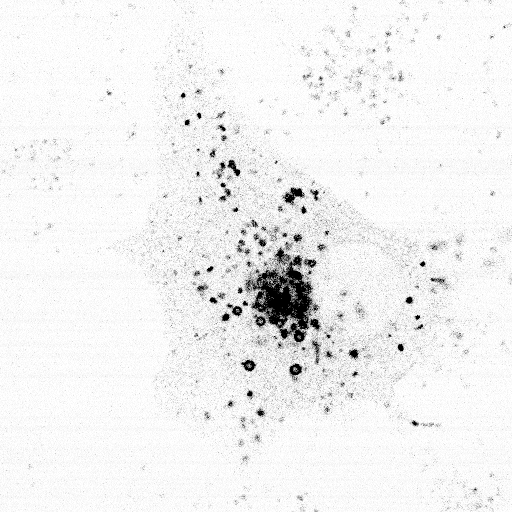

Supplement: S4 File — Raw “tiff” image files of the cell shown in Fig 4. “pooled” is the Graphpad Prism spreadsheet containing normalized intensity and nMDP data from all cells analyzed and plotted in the graph in Fig 4. (ZIP) [file pone.0139957.s004.zip › Fig4/lamp1--2.tif]

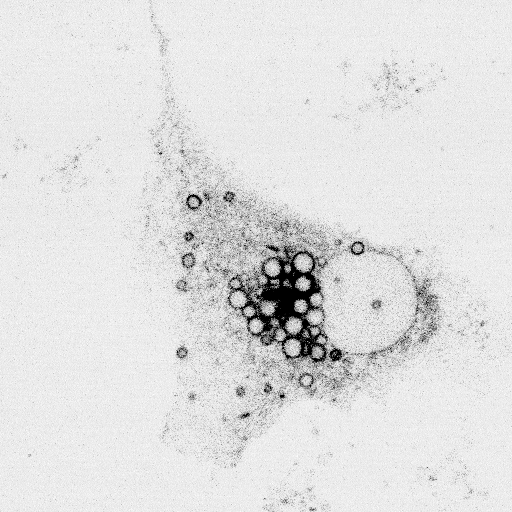

Supplement: S4 File — Raw “tiff” image files of the cell shown in Fig 4. “pooled” is the Graphpad Prism spreadsheet containing normalized intensity and nMDP data from all cells analyzed and plotted in the graph in Fig 4. (ZIP) [file pone.0139957.s004.zip › Fig4/lamp1-180.tif]

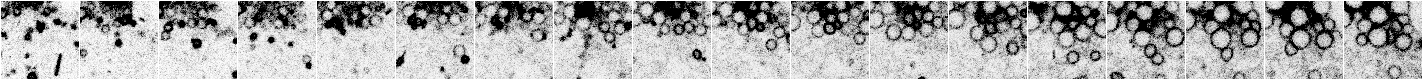

Supplement: S4 File — Raw “tiff” image files of the cell shown in Fig 4. “pooled” is the Graphpad Prism spreadsheet containing normalized intensity and nMDP data from all cells analyzed and plotted in the graph in Fig 4. (ZIP) [file pone.0139957.s004.zip › Fig4/Lamp1-Montage.tif]

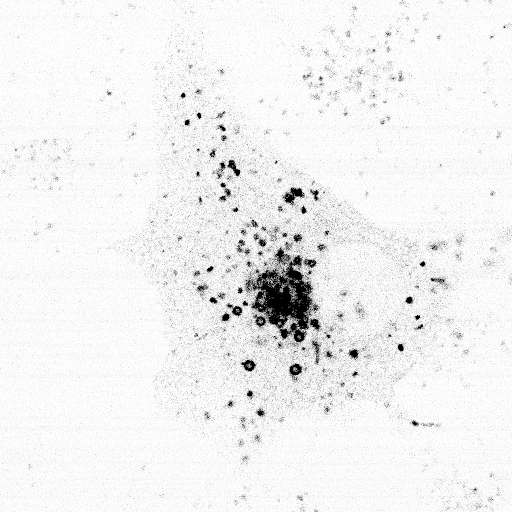

Supplement: S4 File — Raw “tiff” image files of the cell shown in Fig 4. “pooled” is the Graphpad Prism spreadsheet containing normalized intensity and nMDP data from all cells analyzed and plotted in the graph in Fig 4. (ZIP) [file pone.0139957.s004.zip › Fig4/lamp1.tif]

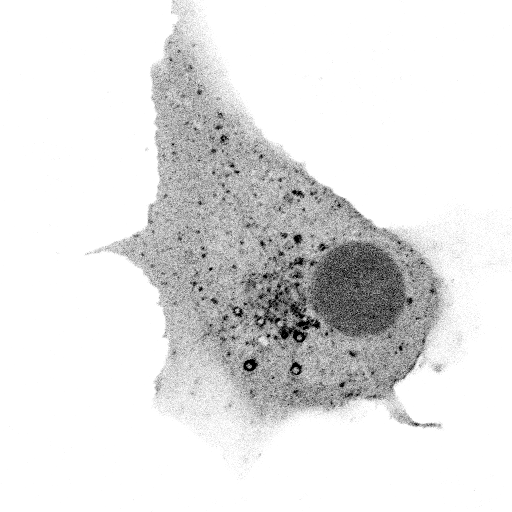

Supplement: S4 File — Raw “tiff” image files of the cell shown in Fig 4. “pooled” is the Graphpad Prism spreadsheet containing normalized intensity and nMDP data from all cells analyzed and plotted in the graph in Fig 4. (ZIP) [file pone.0139957.s004.zip › Fig4/ML1Nx2--2.tif]

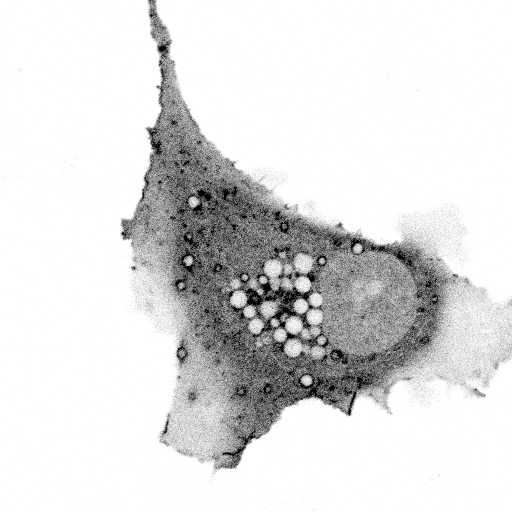

Supplement: S4 File — Raw “tiff” image files of the cell shown in Fig 4. “pooled” is the Graphpad Prism spreadsheet containing normalized intensity and nMDP data from all cells analyzed and plotted in the graph in Fig 4. (ZIP) [file pone.0139957.s004.zip › Fig4/ML1Nx2-180.tif]

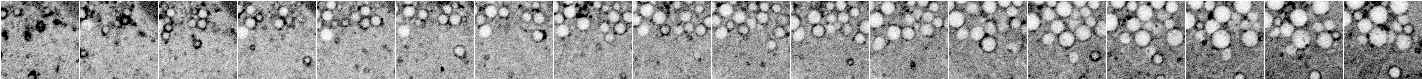

Supplement: S4 File — Raw “tiff” image files of the cell shown in Fig 4. “pooled” is the Graphpad Prism spreadsheet containing normalized intensity and nMDP data from all cells analyzed and plotted in the graph in Fig 4. (ZIP) [file pone.0139957.s004.zip › Fig4/ML1Nx2-Montage.tif]

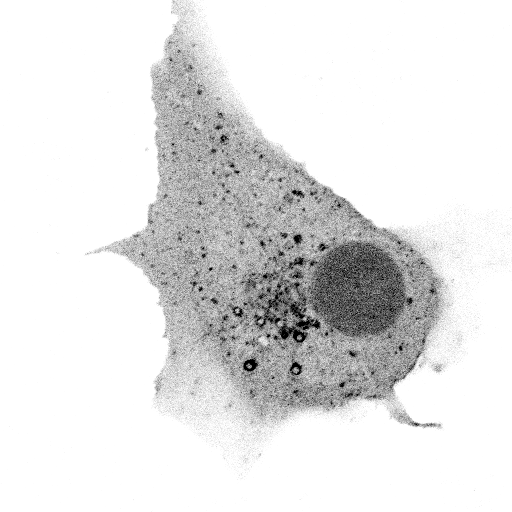

Supplement: S4 File — Raw “tiff” image files of the cell shown in Fig 4. “pooled” is the Graphpad Prism spreadsheet containing normalized intensity and nMDP data from all cells analyzed and plotted in the graph in Fig 4. (ZIP) [file pone.0139957.s004.zip › Fig4/ML1Nx2.tif]

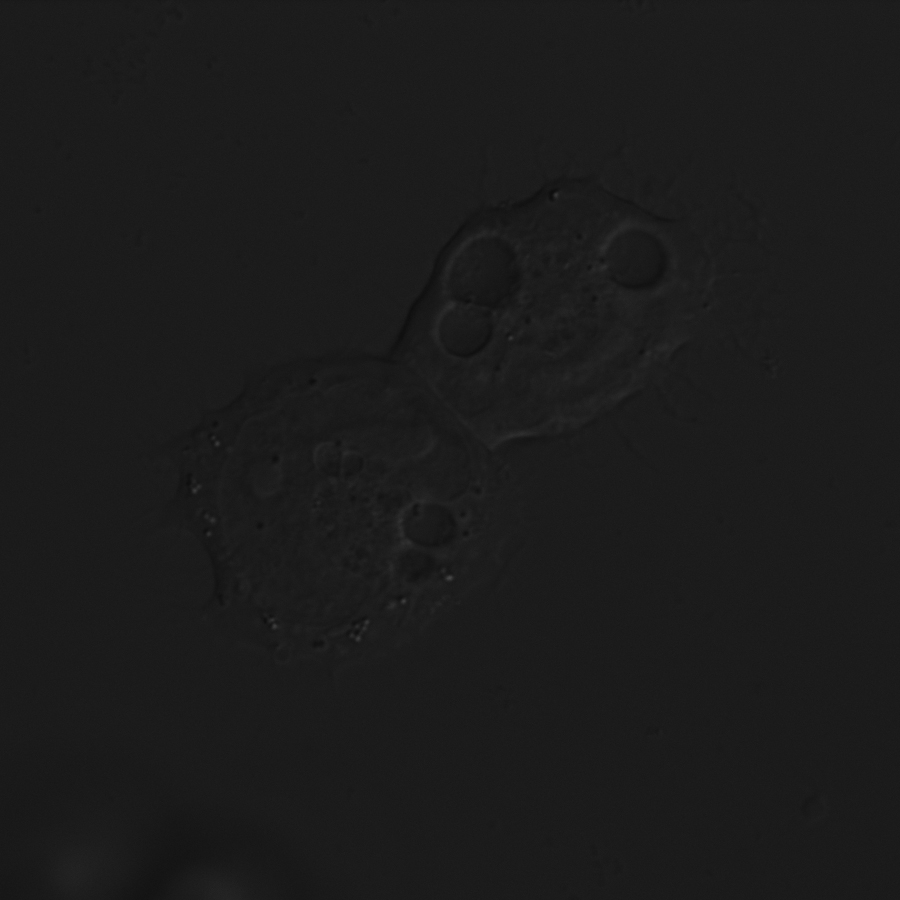

Supplement: S5 File — Raw “jpeg” image files of the cell shown in Fig 4. (ZIP) [file pone.0139957.s005.zip › Fig5/150413 To Gerry/KO1-DIC.jpg]

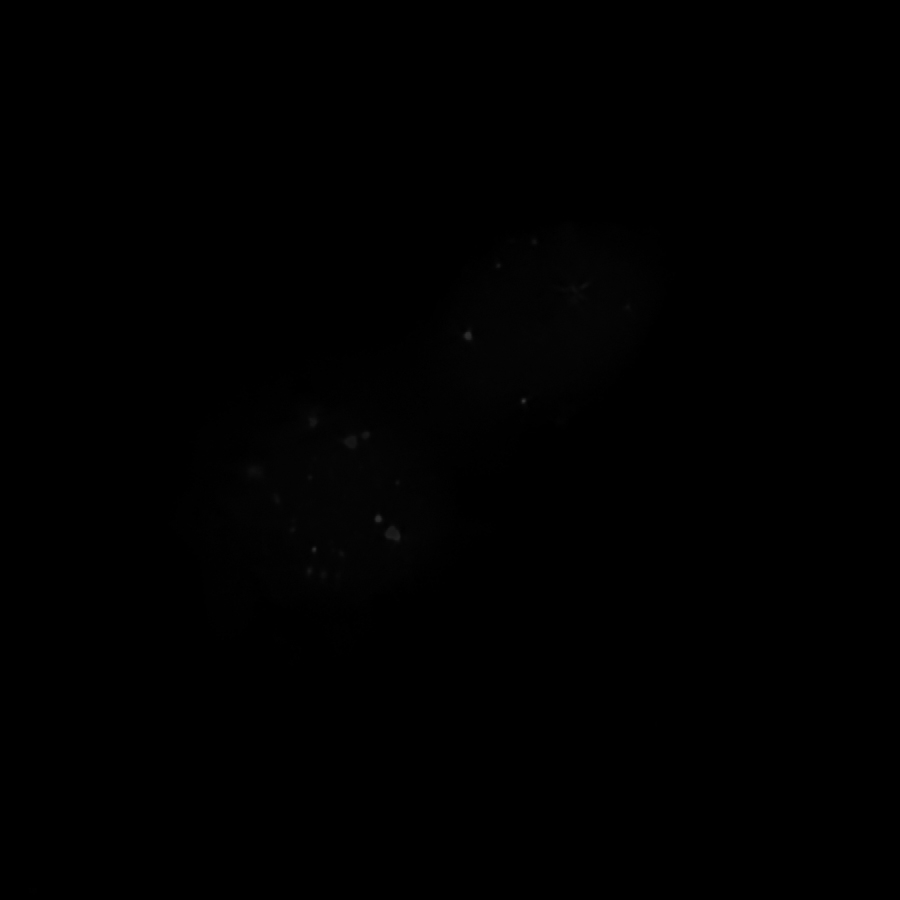

Supplement: S5 File — Raw “jpeg” image files of the cell shown in Fig 4. (ZIP) [file pone.0139957.s005.zip › Fig5/150413 To Gerry/KO1-GFP.jpg]

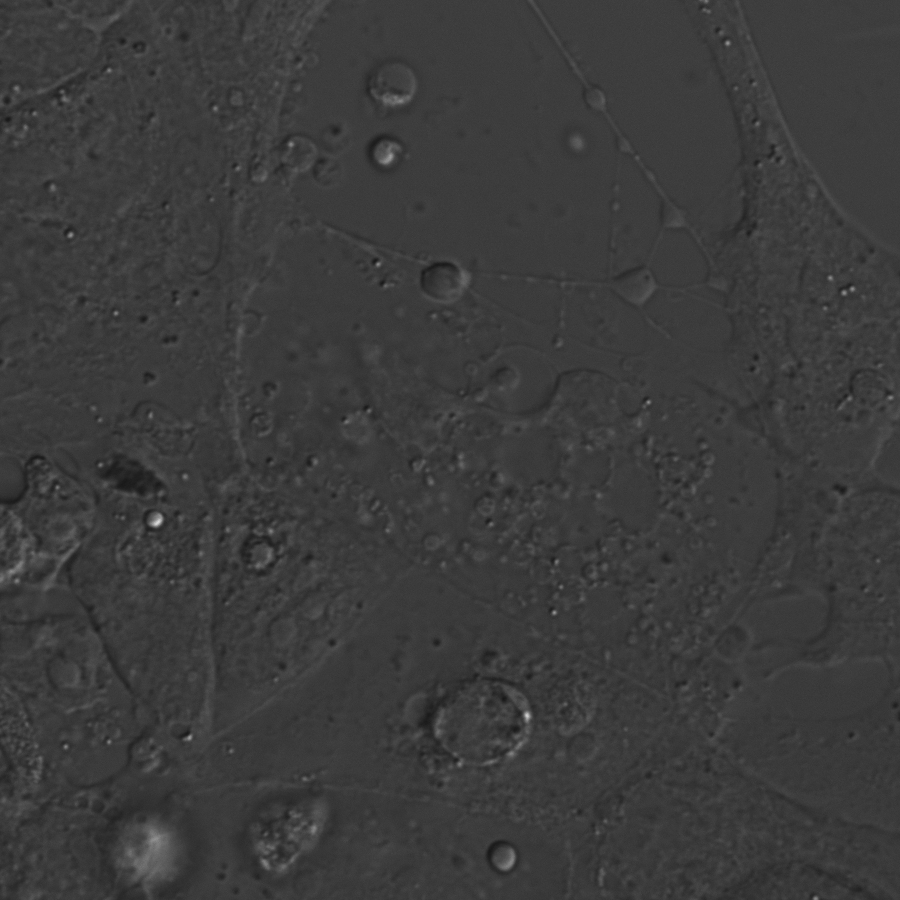

Supplement: S5 File — Raw “jpeg” image files of the cell shown in Fig 4. (ZIP) [file pone.0139957.s005.zip › Fig5/150413 To Gerry/KO2-DIC.jpg]

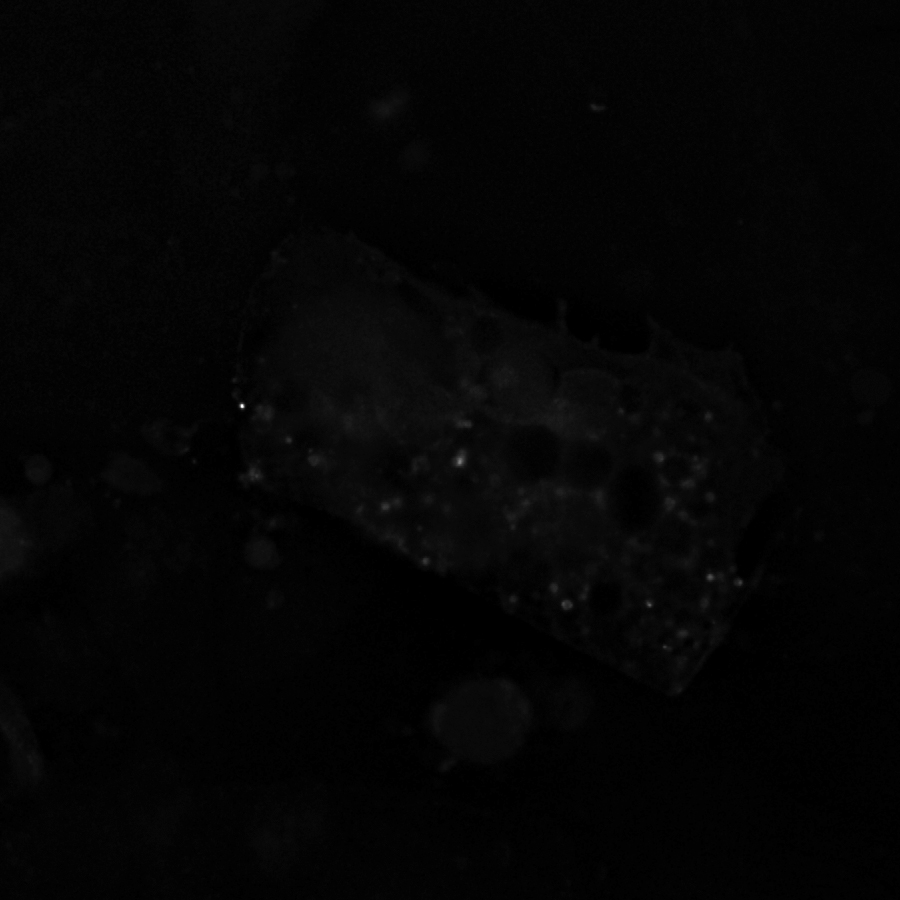

Supplement: S5 File — Raw “jpeg” image files of the cell shown in Fig 4. (ZIP) [file pone.0139957.s005.zip › Fig5/150413 To Gerry/KO2-GFP.jpg]

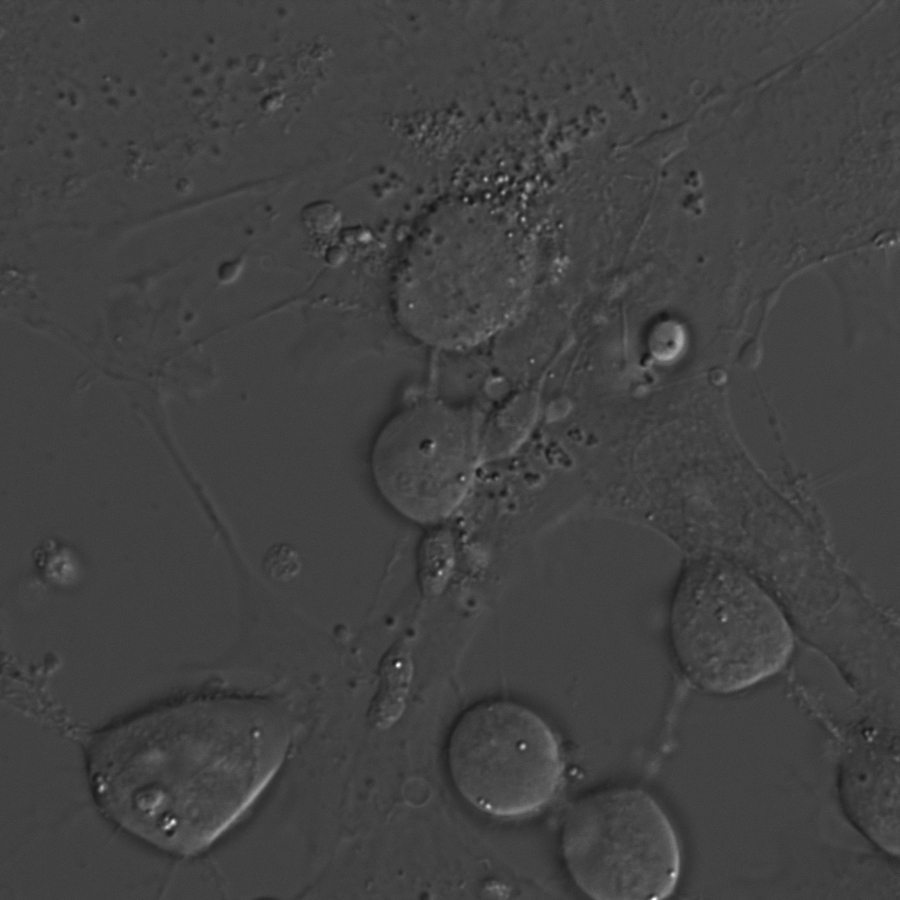

Supplement: S5 File — Raw “jpeg” image files of the cell shown in Fig 4. (ZIP) [file pone.0139957.s005.zip › Fig5/150413 To Gerry/WT-DIC.jpg]

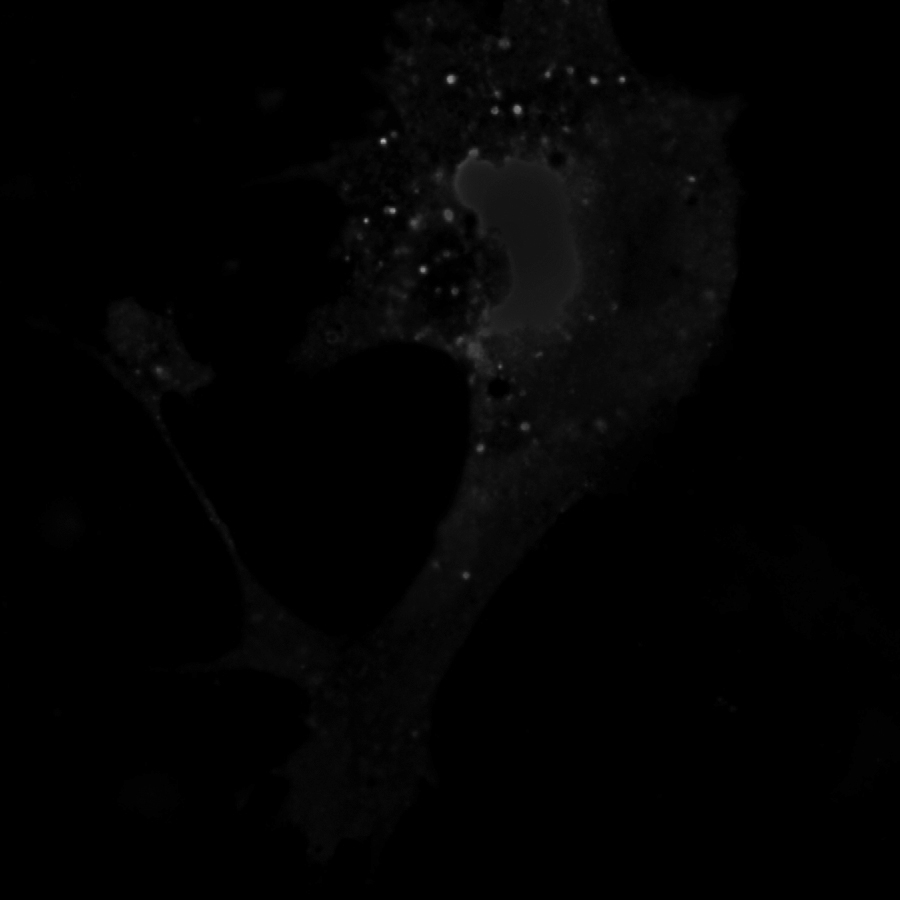

Supplement: S5 File — Raw “jpeg” image files of the cell shown in Fig 4. (ZIP) [file pone.0139957.s005.zip › Fig5/150413 To Gerry/WT-GFP.jpg]

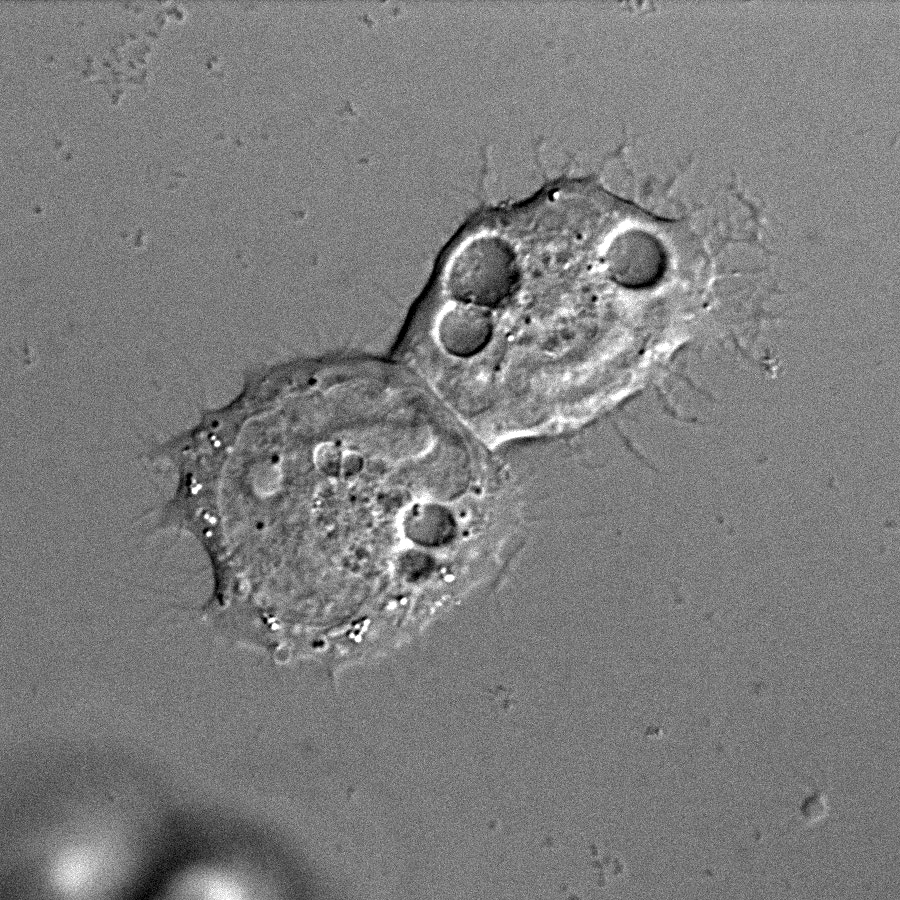

Supplement: S5 File — Raw “jpeg” image files of the cell shown in Fig 4. (ZIP) [file pone.0139957.s005.zip › Fig5/KO1-DIC.jpg]

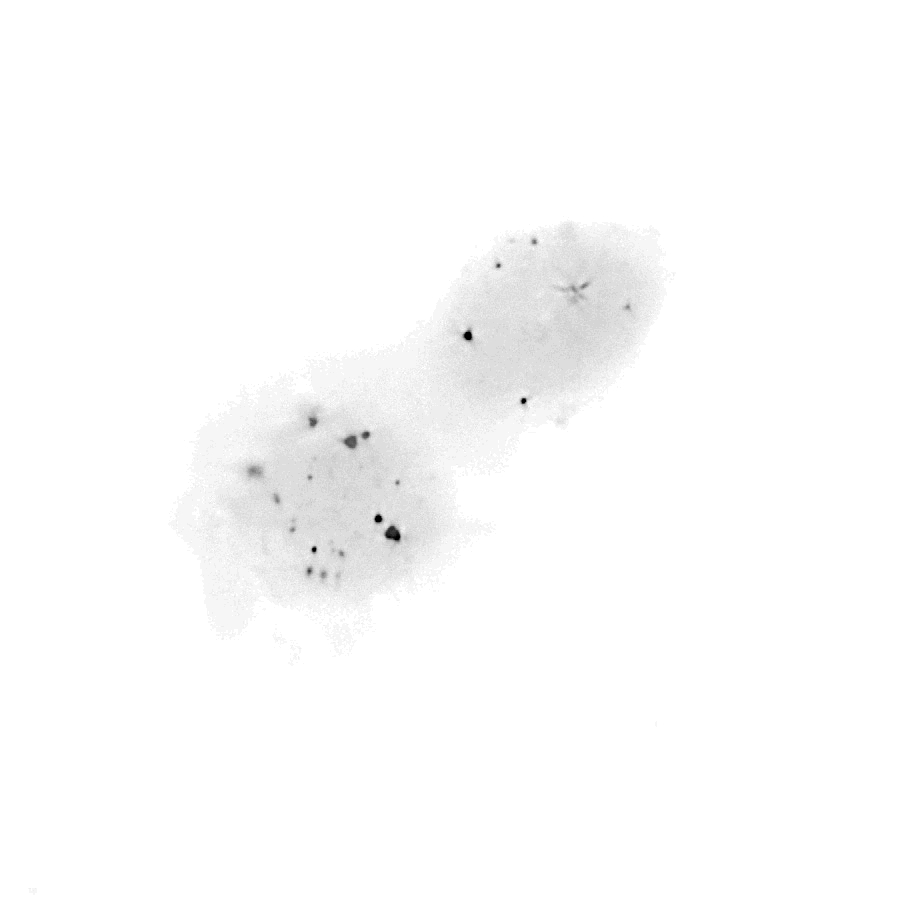

Supplement: S5 File — Raw “jpeg” image files of the cell shown in Fig 4. (ZIP) [file pone.0139957.s005.zip › Fig5/KO1-GFP.jpg]

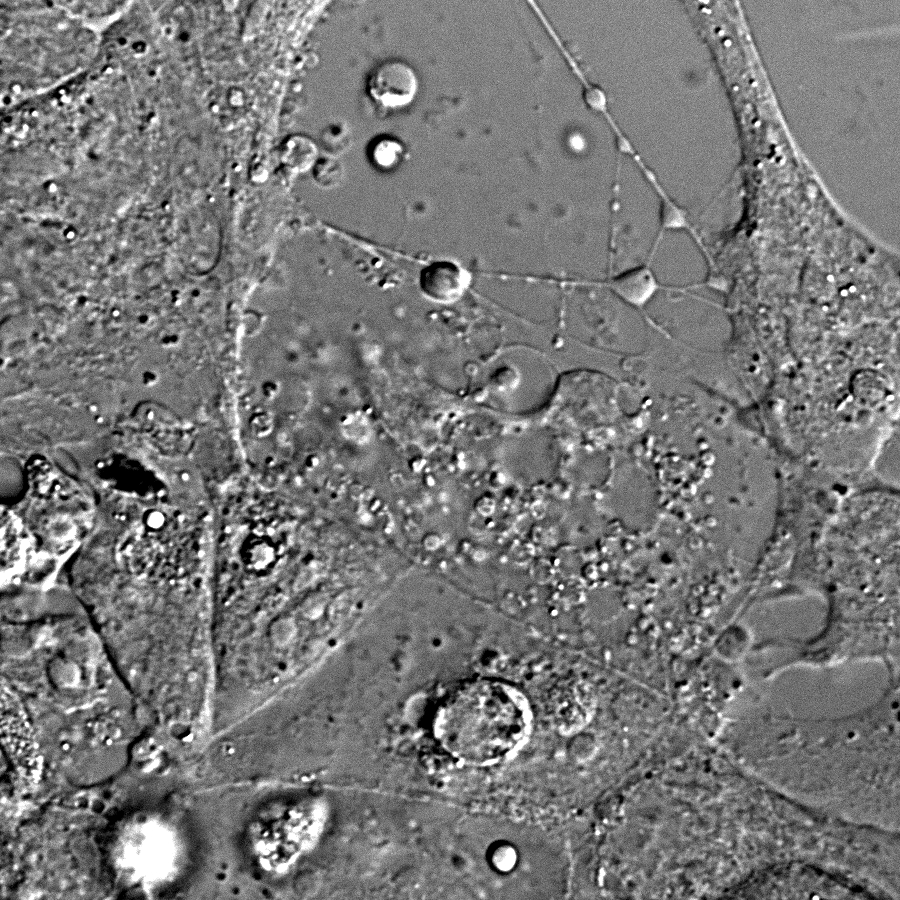

Supplement: S5 File — Raw “jpeg” image files of the cell shown in Fig 4. (ZIP) [file pone.0139957.s005.zip › Fig5/KO2-DIC.jpg]

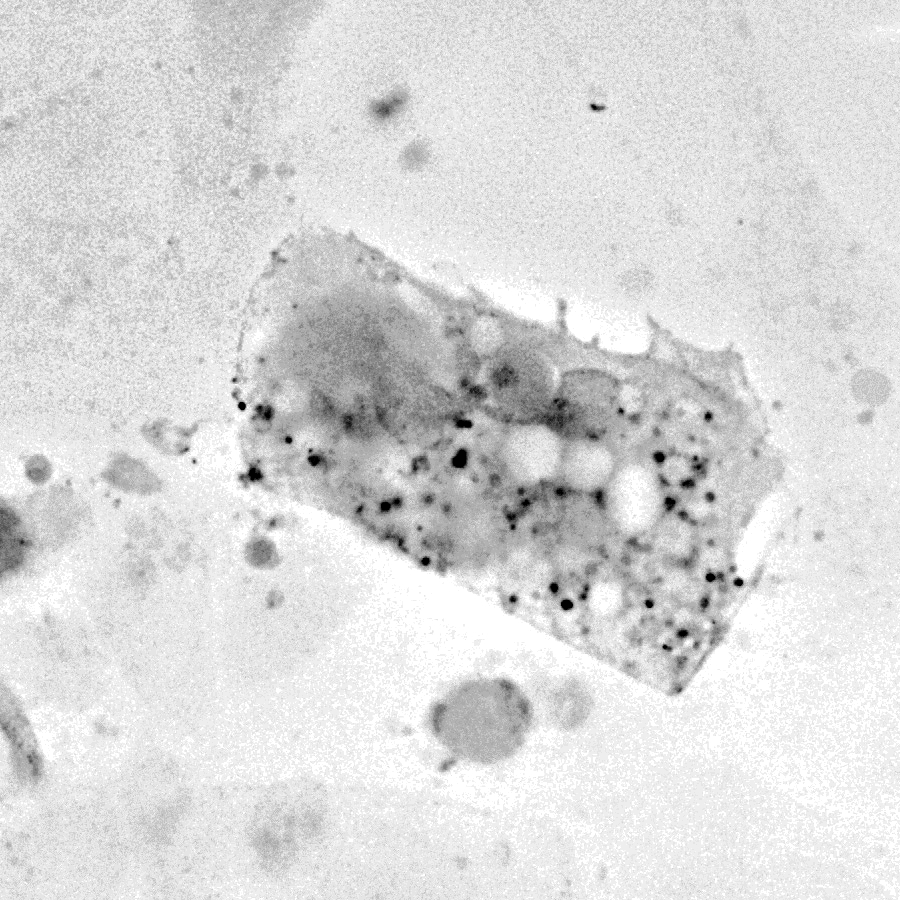

Supplement: S5 File — Raw “jpeg” image files of the cell shown in Fig 4. (ZIP) [file pone.0139957.s005.zip › Fig5/KO2-GFP.jpg]

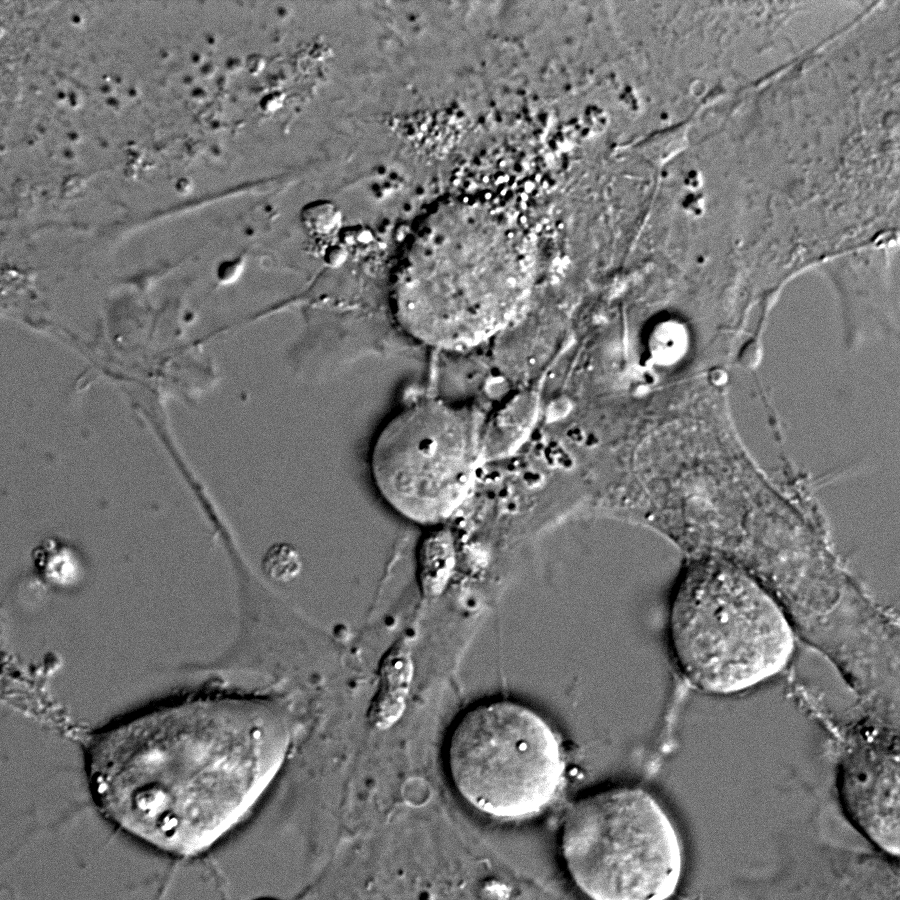

Supplement: S5 File — Raw “jpeg” image files of the cell shown in Fig 4. (ZIP) [file pone.0139957.s005.zip › Fig5/WT-DIC.jpg]

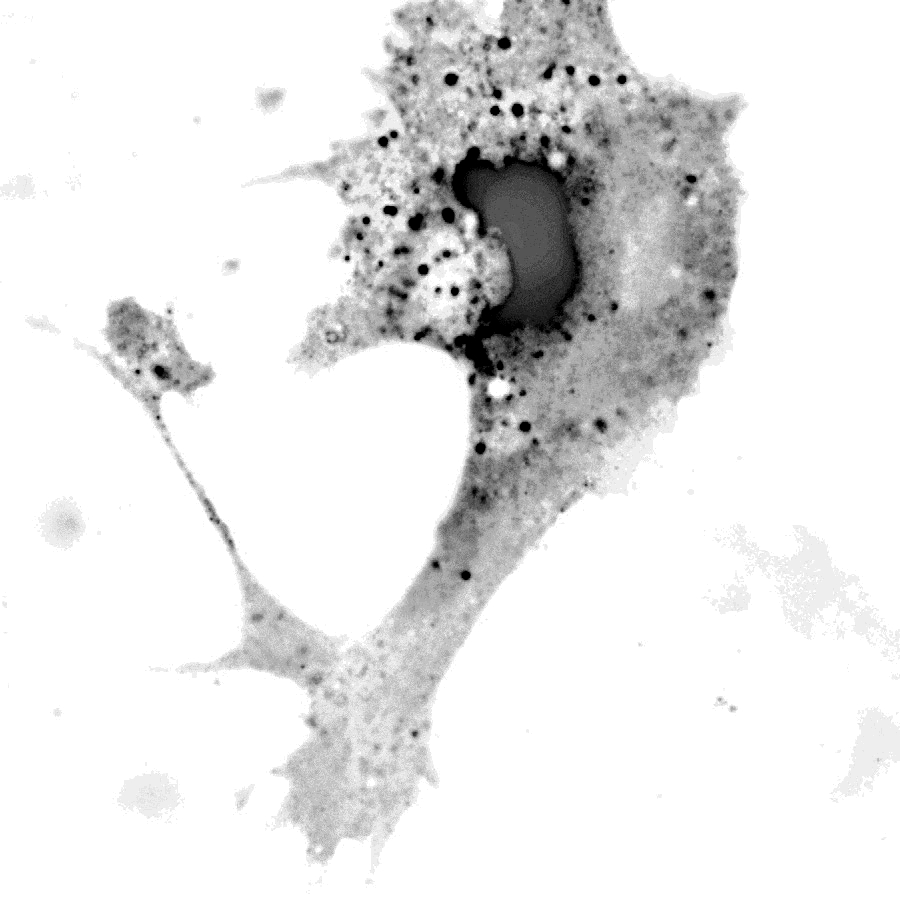

Supplement: S5 File — Raw “jpeg” image files of the cell shown in Fig 4. (ZIP) [file pone.0139957.s005.zip › Fig5/WT-GFP.jpg]
